# The transmission ability in a population of elite tetraploid potatoes

**DOI:** 10.1002/tpg2.70066

**Published:** 2025-06-30

**Authors:** Trine Aalborg, Hélène Romé, Christina Ranzau, Merethe Bagge, Just Jensen, Kåre Lehmann Nielsen

**Affiliations:** ^1^ Department of Chemistry and Bioscience Aalborg University Aalborg Denmark; ^2^ Center for Quantitative Genetics and Genomics Aarhus University Aarhus Denmark; ^3^ Danespo A/S Give Denmark; ^4^ KMC Amba, Denmark Brande Denmark

## Abstract

Crop potato, *Solanum tuberosum* L., has great genetic improvement potential, but vegetative propagation, tetrasomic inheritance, and inbreeding depression have limited genetic gain in the elite gene pool. To elucidate the transmission ability of breeding material in the current elite gene pool, we estimated the additive genetic variance and specific combining abilities among 18 parents for 10 key agronomic traits and evaluated the impact of genotypic information in capturing trait inheritance patterns using genomic prediction. Analysis of 5013 clones from an F_1_ population of an 18‐parent incomplete diallel cross showed that within the population, some traits had greater additive genetic effects, while others had greater nonadditive genetic contributions. There were clear differences in the breeding values between the evaluated parents. The effect of cytoplasmic genome type on phenotype was assessed and compared to its impact on fertility. These statistics were used to discuss the parental potential of the diallel parents. Including genotyping‐by‐sequencing information (available for 15% of the population) in a single‐step approach improved the prediction accuracy of genetic breeding values, and, surprisingly, the greatest effect was observed for the genomic prediction of high‐heritability traits. Increased prediction accuracy across multiple traits is crucial to support the breeder's decisions when selecting parents. The phenotypic and genetic correlations between traits were determined using pairwise bivariate models. Significant genetic correlations between several traits were identified, indicating that single‐trait selection in breeding programs will result in the indirect selection of correlated traits—possibly with opposing additive contributions to different breeding goal traits by the same progenitor.

## Core Ideas

- Cytoplasmic genome type can impact clone fertility and some trait phenotypes and should influence parent selection.
- All traits had significant additive genetic contributions, indicating potential for genetic gain through breeding.
- It was trait dependent whether additive versus nonadditive genetic effects were more important.
- Prediction accuracy of parent estimated breeding values improves with the incorporation of genomic and pedigree information in ssGBLUP.
- Significant pairwise trait genetic correlations indicate extensive indirect trait selection during breeding.

**Abbreviations**
BLUP: best linear unbiased prediction
EBV: estimated breeding value
GCA: general combining ability
GBLUP: genomic BLUP
GBS: genotyping‐by‐sequencing
GWAS: genome‐wide association study
PA: prediction accuracy
PBLUP: pedigree BLUP
PEV: prediction error variance
QTL: quantitative trait locus
SCA: specific combining ability
SNP: single nucleotide polymorphism
ssGBLUP: single‐step GBLUP

## 1 INTRODUCTION

Global food security is facing immediate challenges due to climate change, environmental concerns, increased urbanization, and the need for biodiversity conservation areas, leading to a current exhaustion and expected future loss of arable farmlands (Lenaerts et al., 2019; W. Wu, Yu, et al., 2018). Concurrently, an increase in global food demand of ∼56% from 2010 to 2050 is predicted (Van Dijk et al., 2021) and this necessitates the advancement of agricultural systems to generate increased yields of high‐quality crops while decreasing resource consumption (Devaux et al., 2019). One strategy to attain this is crop improvement by accelerating the breeding of superior crop cultivars (Lenaerts et al., 2019). The potato crop, *Solanum tuberosum* L., is an attractive candidate for breeding optimization strategies since it has a high nutritional value, excellent yield, and global cultivation as a staple food (Devaux et al., 2014). It is currently the world's fourth most important food crop and was in 2022 globally cultivated on 17.8 million ha of cropland, yielding a total production of ∼375 Mt (FAOSTAT, 2024).

Furthermore, the potato is likely to harbor great potential for genetic improvement. The domestic tetraploid potato cultivars, delineated to Andean origin from landrace progenitors (Gutaker et al., 2019; Spooner et al., 2005), constitute a highly heterozygous gene pool of breeding material (Gebhardt, 2016). The overall aim of potato breeding programs is the development of new cultivars for marketing and breeding clones for introduction as new parental lines by crossing breeding material to make seeds with new allele combinations superior to those in either progenitor (Hickey et al., 2017). However, potatoes are vegetatively propagated, which causes varieties to exist indefinitely, and the breeding history of domesticated tetraploid elite potato has many recurrent parents at different levels in the pedigree. Furthermore, there have been relatively few sexual generations during the period of modern breeding starting around 1850s, following its post‐Colombian introduction into Europe around the 1600s (Brown, 1993). Together with the tetrasomic inheritance of potatoes, this has produced only minimal changes to the allele frequencies of the elite gene pool, causing the genetic gain of potato breeding to be low (Ortiz, 2020; Slater et al., 2016).

Breeding programs in potatoes have traditionally relied on phenotypic selection and, more recently, partially on pedigree‐based selection. Also, the advent of affordable genome sequencing technology and high‐throughput genotyping has allowed the incorporation of genetic variants into selection models, termed genomic selection (Meuwissen et al., 2001). Genomic selection is becoming widely employed in potato breeding to increase the genetic gain of key agronomic traits and accelerate breeding by predicting genomic estimated breeding values (genomic EBVs) (Ortiz, 2020), using a variety of statistical and machine‐learning methods (Ortiz, 2020; Ortiz et al., 2023; Sverrisdóttir et al., 2017; Wilson et al., 2021), including the standard additive genomic BLUP (GBLUP, where BLUP is best linear unbiased prediction) model (Meuwissen et al., 2001). Nonadditive genetic effects are lost over sexual generations due to recombination (epistatic effects) and independent assortment of chromosomes (dominance effects). However, they may still be very relevant to select variants for the market if the ratio of nonadditive compared to additive genetic variation is significant because the nonadditive genetic effects are maintained during vegetative propagation. This may be particularly valuable for lowheritability traits where nonadditive effects are generally more important (Endelman et al., 2018; Meuwissen et al., 2001; Wilson et al., 2021). For such traits, traditional phenotype‐directed breeding has been largely ineffective in autotetraploid potato (Wilson et al., 2021). It is thus relevant to elucidate whether this results from additive but complex polygenic inheritance patterns or whether nonadditive mechanisms of inheritance are not accounted for. The estimation of additive genetic effects and specific combining abilities (SCAs) for key agronomic traits can, therefore, impact the design and performance of genomic prediction models. The SCAs are effects that are due to the combined effect of specific parents, whereas the parent general combining ability (GCA) is the general effect of a parent in all potential matings (Gallais, 2003). Additionally, if the SCA is significant, it can impact the cultivars selected for the commercial market, as they can be selected based on total genetic value, whereas parents in the breeding program should be selected for the total additive genetic value because it is preserved across recombination in future generations.

Historically, combining ability studies have been applied in potato research since the 1960s (Plaisted et al., 1962) to evaluate the agronomic trait transmission ability of parental lines, usually using a diallel cross of a selection of clones in the breeding population (Griffing, 1956). Results are specific to the breeding material from which they are estimated (Bradshaw, 2022)—some previous studies have found greater GCA variance compared to SCA for a host of traits, including yield, dry matter content, emergence, maturity, fry color, and tuber count (Bradshaw et al., 2000; Darabad et al., 2020; Ruiz de Arcaute et al., 2022; Terres et al., 2017), while others have found nonadditive contributions to have ascendancy in traits such as tuber shape, count, and weight with fluctuations across generations (Gopal, 1998; Terres et al., 2017). In addition, GCA values are highly differentiated between parents (Ortiz et al., 1988). General combining abilities can be a key parameter in parent selection and are superior compared to traditional phenotypic selection based on mean phenotypic values by accounting for both the efficiently transmitted additive genetic effects and distinguishing this from the nonadditive contribution. However, their widespread implementation by breeders has been limited (Bradshaw, 2022) as accurate selection of parents based on additive genetic value requires the separation of additive and nonadditive effects. However, the separation of the nonadditive effects is only available for existing offspring. Hence, predictors for novel crosses require complex use of genomic information for unbiased results (Chu & Jensen, 2025; Kristensen et al., 2023).

**Core Ideas**

- Cytoplasmic genome type can impact clone fertility and some trait phenotypes and should influence parent selection.
- All traits had significant additive genetic contributions, indicating potential for genetic gain through breeding.
- It was trait dependent whether additive versus nonadditive genetic effects were more important.
- Prediction accuracy of parent estimated breeding values improves with the incorporation of genomic and pedigree information in ssGBLUP.
- Significant pairwise trait genetic correlations indicate extensive indirect trait selection during breeding.

Our interest in revisiting additive genetic variance in potato is to estimate its importance for genomic prediction models. We further explore the differences between parents and segregation within full‐ and half‐sib families using a training population of a diallel cross between parents derived from a representative sample of the germplasms in the elite gene pool of the Danish potato breeding company Danespo A/S. That is, determining the fraction of the total genetic trait variation that can be captured in additive prediction models by estimating the proportion of the variation that is attributable to additive versus nonadditive effects (the SCA/GCA ratio), where the SCA variance is defined as the sum of dominance and epistatic variation due to interaction between parental genomes, and the GCA variance is one‐fourth of the additive genetic variance (Gallais, 2003). Besides the additive and nonadditive genetic contributions, the cytoplasmic genome type (i.e., the genetic variance found in the mitochondrial and plastid genomes) has also been found to contribute to the phenotypic variance of some agronomic traits, for example, tuber shape, vine maturity, starch content, and yield (Sanetomo & Gebhardt, 2015). However, due to the limited study of the effects of cytoplasmic genome type on phenotypes, the cytoplasmic genome effects are only known for a limited array of traits and have not been reproduced in populations including non‐selected clones. Six overall types of potato cytoplasmic genomes have been identified, that is, T, D, W, A, M, and P, some with respective α‐, β‐, and γ‐ mitochondrial subtypes (Lössl et al., 2000; Sanetomo & Gebhardt, 2015). In the European germplasm pool, the types A, M, and P are relatively rare, while T is predominant (Sanetomo & Gebhardt, 2015). Besides contributing to the total genetic variation of cultivars, some of the cytoplasmic genome types can also confer cytoplasmic male sterility, that is, types D and subtype W/γ (Hosaka & Sanetomo, 2012; Lössl et al., 2000; Sanetomo & Gebhardt, 2015). A specific open reading frame encoded in the mitochondrial genome, ORF137, was identified by comparative mitochondrial genome analysis, and genetic variation in the open reading frame is thought to be associated with cytoplasmic male sterility in *S. tuberosum* (Lian et al., 2024). Through their unintentional propagation in the European gene pool, the cytoplasmic male sterility now constitutes a major genetic bottleneck in modern potato breeding programs (Provan et al., 1999). In modern breeding programs, it is relevant to consider the phenotypic contribution of the cytoplasmic genome type in concert with its possible effect on fertility to make informed selection—while commercial clones may possibly benefit from the phenotypic contribution of a male sterile cytoplasmic genome type, retaining fertility is paramount in parent selection.

Another property relevant to evaluating the power of genomic prediction in breeding programs arises from its contextual application. While genomic selection models are commonly optimized for single traits (Aalborg et al., 2024; Stich & Van Inghelandt, 2018; Sverrisdóttir et al., 2017, 2018; Wilson et al., 2021), breeding programs involve the concurrent selection of multiple agronomic performance characteristics, for example, for optimizing the same trait in disparate environments (Ortiz et al., 2023; Pandey et al., 2023) or co‐selecting for several traits relevant for tuber quality, all requiring the application of multi‐environment/multi‐trait prediction models that can account for genetic correlations between the traits and between traits’ expressions in different environments. To guide parental selection on multiple traits, both individual traits’ genetic variance and the genetic and environmental correlations between traits need to be accounted for to estimate a direct selection response in selected traits, as well as indirect responses in correlated traits not under direct selection (Neyhart et al., 2019).

This study aimed to estimate population parameters of 10 key agronomic traits relative to transmission to progeny. We used the MASPOT experimental population, consisting of 5013 clones of an F_1_ population generated from an incomplete diallel cross of 18 elite cultivar/breeding material parents, to investigate the transmitting ability of the 18 parental cultivars included in the experiment. The traits include dry matter content, yield, eye depth, flesh color, skin finish, senescence, tubers/plant, length, diameter, and length/width ratio. The MASPOT experimental population has previously been used in genomic prediction studies of various agronomic traits (Sverrisdóttir et al., 2017), to evaluate the effect of expanding the training population in genomic prediction models (Sverrisdóttir et al., 2018), and to investigate the effect of marker type, ploidy, and density on genomic prediction and genome‐wide association study (GWAS) of tetraploid potatoes (Aalborg & Nielsen, 2024; Aalborg et al., 2024). Here, we used phenotypic data of the MASPOT population and of the F_0_ parents to estimate the amount of total genetic variation attributable to additive genetic effects and SCA. It was also investigated whether the relative amount of additive genetic variance and SCA variance is trait‐dependent and related to trait heritability in the defined population. The effect of the maternally inherited cytoplasmic genome type on trait phenotypes was also assessed relative to the fertility phenotype, also controlled by these genetic elements. Furthermore, we estimated the phenotypic and additive genetic correlations between the traits analyzed.

## 2 MATERIALS AND METHODS

### 2.1 Plant material

**FIGURE 1:**
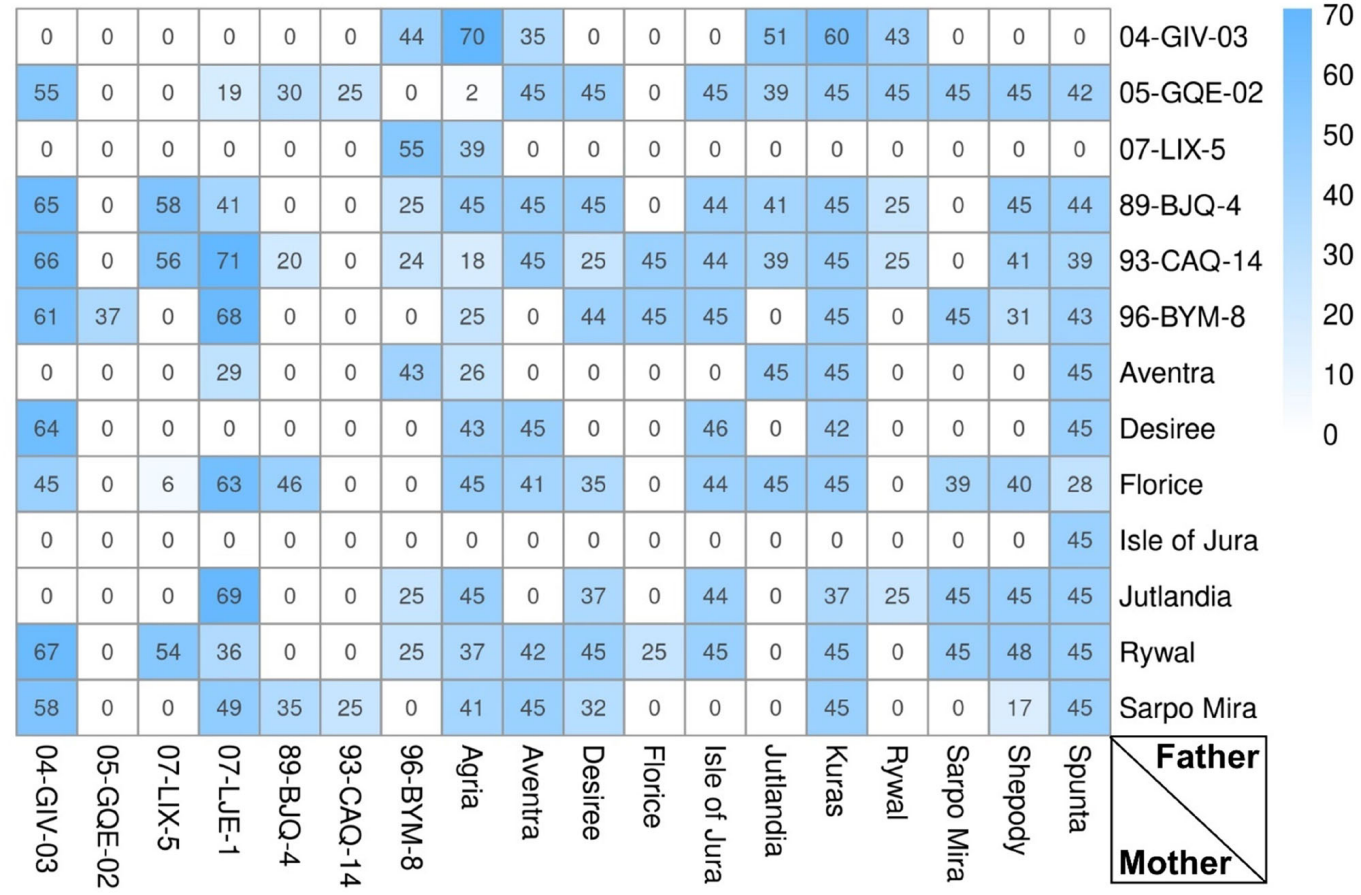
Crossing scheme of the MASPOT incomplete diallel cross showing the number of full‐sibs generated within each family in the F_1_ generation in the full population of 5013 clones.

The MASPOT population, consisting of 5013 F_1_ clones from an incomplete diallel cross of 18 parents (Figure 1), was created by Danespo A/S. The 18 elite parents, either established cultivars or advanced breeding clones from the Danespo A/S breeding program, were selected to be relatively unrelated through pedigree (maximum 12.5%) (Figure S1). The MASPOT parents belonged to different market segments, that is, fresh market, starch, and processing (French fry and crisps). The population was generated by systematic cross‐pollination of all parent combinations, and all vegetative progeny plants capable of producing at least three tubers/plant were propagated and phenotyped for 10 agronomic traits. The diallel crossing design was limited by the male sterility of five parent cultivars and the low fertility of specific crosses, either failing to generate successful seeds or producing seeds that germinated into degenerate and/or non‐tuberizing plants. The pedigree inbreeding coefficient of the full MASPOT population was found to be 0.00021, and the genomic inbreeding coefficient for a representative panel of 755 genotyped clones was 0.033.

The F_1_ clones were planted in field trials in Vandel, Denmark, in 2013 and 2014, as previously described (Aalborg et al., 2024; Sverrisdóttir et al., 2017). Tuber seedlings were planted in 24‐parcel blocks (no replicates and no checks) in 2013 and in randomized 28‐parcel blocks (two replicates, using a single check) in 2014. The parents were also planted in replicates in 2014 and functioned as checks. Plants were grown with a density of approximately 40,000 plants/ha, with 30 cm between plants and 75 cm between rows. In 2014, the plants were grouped by senescence to minimize “neighbor vigor effects” in the trials. Propagation was conducted concurrently with trials, and hence, the number of tubers available in 2013 was lower compared to 2014.

### 2.2 Cytoplasm genome type

The effect of cytoplasm type on trait phenotype was included in all models. Cytoplasm types were assigned based on trace‐back of the maternal pedigree, using the Danespo A/S breeding database of pedigree on the breeding materials or the WUR Potato Pedigree Database (Hutten & Van Berloo, 2001; Van Berloo et al., 2007) for established cultivars, until a maternal progenitor was encountered with cytoplasm type determined by Sanetomo and Gebhardt (2015). For cultivars of non‐European maternal descent, cytoplasm types determined by Danespo A/S were used.

### 2.3 Phenotypes

A total of 10 traits were phenotyped, namely dry matter content, yield, eye depth, flesh color, skin finish, senescence, tubers/plant, length, diameter, and length/width ratio.

#### 2.3.1 Dry matter content

Dry matter content (%) was determined for clones harvested in 2013 (one replicate) and 2014 (two replicates). Tubers were washed, and a basket holding 1.5–10 kg of tubers was weighed above and below water shortly after harvest. Dry matter content was computed using an empirical equation (Equation 1) modified from the Haase (2003) equation for dry matter content estimation from specific gravity based on best linear fit with Danespo A/S data:

(1) $$\begin{array}{ccc} \mathrm{DM}\;\left(\%\right) & = & 214\times (\left(\frac{\mathrm{Weight}\;\mathrm{in}\;\mathrm{air}}{\left(\mathrm{Weight}\;\mathrm{in}\;\mathrm{air}\right)-\left(\mathrm{Weight}\;\mathrm{in}\;\mathrm{water}\right)}\right)\\ & & -\;0.988) \end{array}$$

where DM is dry matter content.

#### 2.3.2 Yield and tuber count

The yield was measured for the two replicates harvested in 2014 as the total weight of five tubers from each clone in two replicates, that is, 2 × 5 tubers for each clone. The weight was converted into hkg/ha, assuming 40,000 plants/ha. The total number of tubers per plant was counted for each clone.

#### 2.3.3 Eye depth

Eye depth was scored manually on a 1–9 scale from deep eyes to no eyes only in 2013. Due to limited application of the 1–4 end of the scale, the phenotype data were recoded to a 1–6 scale by pooling all observations ≤4 and resetting the scale to deep = 1.

#### 2.3.4 Flesh color

Flesh color was scored manually on a 1–9 scale from white to orange flesh for the 2014 harvested clones.

#### 2.3.5 Skin finish

Skin finish was scored manually on a 1–9 scale from rough to smooth for the 2014 harvested clones. Due to the limited application of the 1–4 end of the scale, the phenotype data were recoded to a 1–6 scale by pooling all observations ≤4 and resetting the scale to rough = 1.

#### 2.3.6 Senescence

Senescence was scored manually on a 1–9 scale from no senescence to late senescence for both the 2013 and 2014 harvested clones. The 2013 measures were used to group the clones according to senescence in the 2014 field layout. The scoring was performed at three temporal points, splitting the scale accordingly: first scoring (when the first cultivars begin dying off) used the upper end of the scale (9‐8‐7), second scoring used the middle of the scale (6‐5‐4), and the third scoring (capturing late senescence cultivars) used the low end of the scale (3‐2‐1). Each clone was only scored once; that is, different plants were scored at each temporal point.

#### 2.3.7 Tuber size and shape

A SCOUT camera (Nextec A/S, No.: 0213) was used to measure whole‐tuber length and diameter for the clones harvested in 2014. Tuber length was defined as the longest measure (mm), and the diameter (width) (mm) as the measure perpendicular to this. These measures did not consider tuber anatomy, where length is defined as the distance from the rose (apex) to the heel (attachment of stolon). Only irregular tubers will violate the true definition, which are relatively rare and assumed to not significantly affect downstream analyses. Outliers were identified on the length or diameter parameters relative to the nearest neighbor and removed following Dixon's *Q*‐test (Dean & Dixon, 1951) before calculation of a length/width ratio, where the critical confidence level, $Q_{\mathrm{crit}}$, was estimated for batches of up to 200 tubers by regression and used for a two‐tailed test as outlined by Rorabacher (1991).

### 2.4 Statistical modeling

All models were fitted using the DMU software package (v6, release 5.6) ( Madsen et al., 2014). Data analysis and other statistical analyses were performed in R Statistical Software (v4.2.2) (R Core Team, 2023) in RStudio (v2024.04.0+735) (Posit team, 2023). All graphics were generated in R using base R or the ggplot2 package (v3.4.2) or (v3.5.1) (Wickham, 2016), unless otherwise stated.

### 2.5 Estimation of variance components and prediction of breeding values

Additive and nonadditive genetic variance components were estimated, and breeding values were predicted for single traits using four models, each following the same general linear mixed model (Equation 2):

(2) $$y=Xb+Z_{a}a+Z_{a}l+Z_{f}f+e,$$

where ***y*** is a vector of phenotypes, ***X*** is the design matrix of the fixed effects, and ***b*** is a vector of fixed effects, including block‐by‐year, to correct for soil heterogeneity and across‐year environmental effects for multi‐year traits, and cytoplasm type; ***a*** is a vector of additive genetic effects (breeding values) with $a\;∼\;N(0,M\sigma_{a}^{2})$, where ***M*** is the relationship matrix and $Z_{a}$ is the incidence matrix relating observations to clones, that is, both additive genetic effects as well as residual line effects. Different assumptions were made depending on the relationship matrix used for the model. ***l*** is a vector of line effects with $l\;∼\;N(0,I_{l}\sigma_{l}^{2})$, where ***I_l_*** is an identity matrix with the number of lines as dimensions; $Z_{f}$ is the design matrix for the full‐sib family (nonadditive) genetic effects due to interactions between parental genomes, indicating sire‐dam subgroups; ***f*** is a vector of the family genetic effects due to interaction between sire and dam contributions with $f\;∼\;N(0,I_{f}\;\sigma_{f}^{2})$, where ***I_f_*** is an identity matrix with the number of families as dimensions; ***e*** is a vector of residuals with $e\;∼\;N(0,I\sigma_{e}^{2})$ with identity matrix ***I*** of dimension number of observations. We estimate the total additive genetic effect of a parental clone (the EBV) and not the classical GCA of parents, which is only half the additive genetic value (EBV = 2GCA). The use of full relationship matrices among all clones allows the exploitation of all relationships in estimating additive genetic variance and in the prediction of breeding values. Only dry matter content relied on phenotype recordings across two years for model fitting, and for this trait the line effect was nested within year of test. The line effect was only included for traits where the clones had replicates; that is, it was excluded for models on eye depth. The variance due to line effects will include nonadditive genetic variances due to interactions between loci inherited from individual parents as well as line‐related effects due to common source and storage of the seed potatoes for each clone. Due to this ambiguity, we do not estimate broad‐sense heritability in this analysis. The SCA/GCA ratio was estimated as the $\frac{\sigma_{f}^{2}}{\frac{1}{4}\sigma_{a}^{2}}$, as the additive value that is transmitted from parent to offspring is half the additive parent value, and the GCA variance equals $\frac{1}{4}\sigma_{a}^{2}$. The incomplete nature of the diallel cross precluded estimation of separate maternal and paternal nonadditive genetic variance components. Instead, the nonadditive genetic variation was estimated as the family variance.

Variance components were estimated for additive genetic variance and SCA (nonadditive genetic variance due to interaction between parental genomes) using four models, namely, pedigree BLUP (PBLUP) on the full MASPOT population (PBLUP_pop_) and on a genotyped subset of 755 clones, called the MASPOT panel (PBLUP_panel_), genomic BLUP on the genotyped panel (GBLUP), and single‐step BLUP (ssGBLUP) on the full MASPOT population. Distinct relationship matrices were used for each model to describe the variance–covariance structure.

#### 2.5.1 PBLUP

A pedigree relationship (***A***) matrix (Figure S2) was constructed from the pedigree information of the MASPOT F_1_ offspring, the MASPOT parents, and the MASPOT grandparent generation for PBLUP for both the full MASPOT population and the genotyped MASPOT panel. The AGHmatrix package in R (v.2.1.4) (Amadeu et al., 2023) with a ploidy of 4 and no double reduction was used to compute the matrices. A fixed level of double reduction was not included, despite it being an established inheritance mechanism in polysomic species (Bourke et al., 2018; Gerard, 2023; Hardy, 2016; Parisod et al., 2010), as simulations have shown low rates of double reduction, with variable rates relative to the distance to the centromeres in tetraploid potatoes (Bourke et al., 2015). Analysis of model fit measured by −2log(L), where *L* is the likelihood of the model, showed a local minimum with 0% double reduction (Figure S3), which therefore was implemented in all models. For the models based on the full and reduced pedigree relationship matrices (PBLUP_pop_ and PBLUP_panel_, respectively), the vector of additive genetic effects is defined as $a\;∼\;N(0,A\sigma_{a}^{2})$. The pedigree inbreeding coefficient was calculated as $\frac{\mathrm{mean}\;(\mathrm{diag}(A))\;-\;1}{\mathrm{ploidy}\;-\;1}$, where diag(***A***) is the diagonal elements of ***A*** for the full MASPOT population (Gallais, 2003).

#### 2.5.2 GBLUP

For GBLUP, genotyping‐by‐sequencing (GBS) allele frequency genotypic data for 755 MASPOT offspring and of the 18 parents were used (reduced crossing scheme in Aalborg et al. [2024]). A previously published 93,170 SNP (single nucleotide polymorphism) dataset filtered to minor allele frequency > 1%, read depth > 5 and < 60, and missing rate < 50% of approximately equal parts non‐synonymous, synonymous, and non‐coding biallelic variants (Aalborg et al., 2024) was used. A genomic relationship (***G***) matrix (Figure S4) was computed using the AGHmatrix package using the VanRaden method for non‐integer dosage genotypes with adjustment for tetraploidy (VanRaden, 2008) and using mean imputation of missing SNP information (17.06% of all markers). For the GBLUP model, the vector of additive genetic effects is defined as $a\;∼\;N(0,G\sigma_{a}^{2})$. The genomic inbreeding coefficient was calculated as $\frac{\mathrm{mean}(\mathrm{diag}(G))-1}{\mathrm{ploidy}\;-1}$, where diag(***G***) is the diagonal elements of ***G*** (Gallais, 2003).

#### 2.5.3 ssGBLUP

For ssGBLUP, a single‐step covariance (***H***) matrix (Figure S5) was constructed by combining the ***A*** and ***G*** matrices using the AGHmatrix package. The scale of the ***G*** matrix was adjusted to the scale of the pedigree relationship matrix of the genotyped clones (***A*_22_**) prior to the combination using Equation (3):

(3) $$G_{\mathrm{adj}}=\frac{G}{\mathrm{mean}\left(\mathrm{diag}\left(G\right)\right)}\times mean\left(\mathrm{diag}\left(A_{22}\;\right)\right).$$

Following adjustment, the matrices were combined using the Martini method (Martini et al., 2018) with τ=ω=1, corresponding to the scaling in Legarra et al. (2009) and Christensen and Lund (2010). For the ssGBLUP model, the vector of additive genetic effects is defined as $a\;∼\;N(0,H\sigma_{a}^{2})$.

#### 2.5.4 Heritability

The narrow‐sense additive genetic heritability was computed as the ratio of additive genetic ($\sigma_{a}^{2}$) to total phenotypic variance ($\sigma_{p}^{2}$) based on the PBLUP_pop_ linear mixed model following Equation (4):

(4) $$\begin{array}{ccc} h^{2} & = & \frac{\left(\sigma_{a}^{2}\right)}{\left(\sigma_{p}^{2}\right)}\\ \sigma_{p}^{2} & = & \sigma_{a}^{2}+\sigma_{f}^{2}+\sigma_{l}^{2}+\sigma_{e}^{2}, \end{array}$$

that is, the heritability of an individual plot record.

#### 2.5.5 Cross‐validation

To validate models, breeding values were predicted using 30 repeats of eightfold random cross‐validation, splitting the lines into folds of equal size. Variance components were estimated in the full dataset using the DMU AIREML software and used as priors for predicting breeding values with DMU4 software, using each fold in each repeat in turn as the validation set and the remaining folds as a reduced training set. Prediction correlation of one repeat was determined as the Pearson correlation between the predicted breeding value of each line in the validation set and the mean corrected phenotype for each line ($\bar{y_{c}}$) (Equation 5). The mean corrected phenotype was calculated by adjusting observed phenotypic values for fixed effects and subsequently calculating the average corrected phenotypic value for each entry. This was done for each of the applied models, using the estimated fixed effects for the respective model in which correlation was determined.

(5) $$r\left(\mathrm{EBV}:\bar{y_{c}}\right).$$

Prediction accuracy (PA) of breeding values was determined as the prediction correlation divided by the square root of the design heritability (Equation 6), where no. rep. designates the number of replicates of each clone:

(6) $$\begin{array}{ccc} \mathrm{PA} & = & \frac{r\left(\mathrm{EBV},\bar{y_{c}}\right)}{\sqrt{h_{\bar{y_{c}}}^{2}\;}}\;,\\ h_{\bar{y_{c}}}^{2} & = & \frac{\sigma_{a}^{2}}{\sigma_{a}^{2}+\sigma_{f}^{2}+\sigma_{l}^{2}+\frac{\sigma_{e}^{2}}{\mathrm{no}.\;\mathrm{rep}.}}. \end{array}$$

The design heritability, accounting for the specific experimental design, was used and calculated based on the variance components of the respective model used to calculate the breeding values.

Dispersion bias was determined as the linear regression coefficient between the mean predicted breeding values (EBVs) and the mean corrected phenotype ($\bar{y_{c}}$). A slope (*β*) = 1 indicates no over‐ or under‐dispersion, while *β* > 1 indicates that extreme EBVs are deflated, and conversely for *β* < 1 (Luan et al., 2009).

#### 2.5.6 Phenotypic and additive genetic correlations

Pairwise phenotypic and additive genetic correlations between traits were estimated using bivariate linear mixed models, using the following model structure for PBLUP_pop_ (Equation 7):

(7) $$\begin{array}{ccc} \left(\begin{array}{c} y_{1}\\ y_{2} \end{array}\right) & = & \left(\begin{array}{cc} X_{1} & 0\\ 0 & X_{2} \end{array}\right)\left(\begin{array}{c} b_{1}\\ b_{2} \end{array}\right)+\left(\begin{array}{cc} Z_{a1} & 0\\ 0 & Z_{a2} \end{array}\right)\left(\begin{array}{c} a_{1}\\ a_{2} \end{array}\right)\\ & & +\left(\begin{array}{cc} Z_{a1} & 0\\ 0 & Z_{a2} \end{array}\right)\left(\begin{array}{c} l_{1}\\ l_{2} \end{array}\right)+\left(\begin{array}{cc} Z_{f1} & 0\\ 0 & Z_{f2} \end{array}\right)\left(\begin{array}{c} f_{1}\\ f_{2} \end{array}\right)\\ & & +\left(\begin{array}{c} e_{1}\\ e_{2} \end{array}\right), \end{array}$$

where ***y*_1_** and ***y*_2_** are the trait 1 and 2 phenotype vectors, ***b*_1_** and ***b*_2_** are the fixed effect vectors for traits 1 and 2 with respective design matrices ***X*_1_** and ***X*_2_**, and ***Z*_a1_** and ***Z*_a2_** are the incidence matrices relating observations to clones. The vector of additive effects $\left(\begin{array}{c} a_{1}\\ a_{2} \end{array}\right)$ follows a distribution of N(0,A⊗C), where ***A*** is the ***A***‐matrix and ***C*** is the variance and covariance matrix of the breeding values of traits 1 and 2. ⊗ is the Kronecker product, so the resulting matrix will have dimensions of the number of individuals in the pedigree by the number of traits in the analysis, that is, 2; ***l*_1_** and ***l*_2_** are the line effects; ***Z*_f1_** and ***Z*_f2_** are the design matrices of the family effect relating ***f*_1_** and ***f*_2_**, the vectors of family effects, to the observations; $\left(\begin{array}{c} e_{1}\\ e_{2} \end{array}\right)$ is the vector of residuals for both traits, following distribution N(0,I⊗R), where ***I*** is an identity matrix and ***R*** is the residual variance and covariance matrix of the traits. While the additive effect incidence matrices were identical for both traits in all pairings, dry matter content again included nested line effects. The line effect was only included for traits with replicate phenotypic observations.

The phenotypic correlation between trait pairs was computed based on the phenotypic covariance matrix for the random effects and residuals, following Equation (8):

(8)

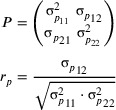




Note that due to eye depth being recorded in 2013 only, and the remaining traits except dry matter content in 2014 only, a residual covariance could not be established for those combinations including eye depth, and hence a phenotypic correlation is not included for those pairs.

The additive genetic correlation between two traits was determined based on the genetic covariance matrix of the genomic breeding values of the two traits (Equation 9):

(9)

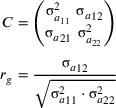




## 3 RESULTS AND DISCUSSION

### 3.1 Phenotypes

**FIGURE 2:**
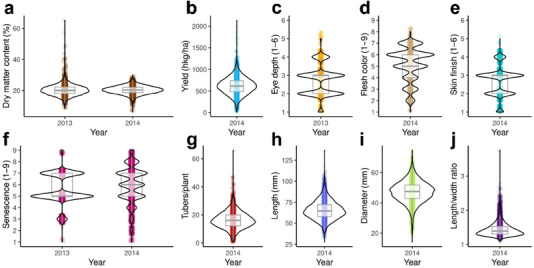
Distribution of phenotypes in the MASPOT population. (a) Dry matter content, (b) yield, (c) eye depth, (d) flesh color, (e) skin finish, (f) senescence, (g) tubers/plant, (h) length, (i) diameter, and (j) length/width ratio.

Phenotypes were recorded for a total of 10 agronomic traits: dry matter content, yield, eye depth, flesh color, skin finish, senescence, tuber count (tubers/plant), tuber length, diameter, and length/width ratio (Figure 2, Table 1). Phenotyping was performed across two consecutive years for dry matter content and senescence. The initial senescence gradings of 2013 were used to group the plants into three groups according to senescence in the 2014 field layout. Only the 2014 phenotypes were used for modeling senescence because neighbors were not conserved across the years 2013 and 2014, and the full grading scale was not applied in 2013 (Figure 2f). In 2013, extreme dry matter contents (>40%) were measured for a small number of cultivars of diverse familial backgrounds and sizes. Some of these cultivars had non‐extreme dry matter content measurements in 2014, while others had been removed from the population due to degeneration (minimum criterion of three tubers/plant each year). Therefore, the extreme 2013 measures were likely a result of measuring error, but in lieu of specific observations that would merit the removal of individual outliers, they were retained in the analysis for dataset completeness.

**TABLE 1:** Statistics describing phenotypic data across years: range, mean ± standard error (SE), phenotypic variance, coefficient of variance (CV), number of observations, and number of replicates (descriptive statistics of the mean corrected phenotypes across years are presented in Table S1).

Phenotype	Range	Mean (± SE)	Variance	CV	No. of observation	No. of replications
Dry matter content (%)	6.00–65.30	20.26 ± 0.03	9.46	0.15	13,481	3
Yield (hkg/ha)	17.00–2136.00	616.71 ± 2.10	37,619.65	0.31	8562	2
Eye depth (1–6)	1–5	2.68 ± 0.01	0.65	0.30	4914	1
Flesh color (1–9)	1–8	5.17 ± 0.02	2.11	0.28	8551	2
Skin finish (1–6)	1–5	2.66 ± 0.01	0.62	0.30	8560	2
Senescence (1–9)	1–9	6.01 ± 0.02	2.21	0.25	8570	2
Tubers/plant	1.00–66.00	16.20 ± 0.06	33.65	0.36	8524	2
Length (mm)	27.67–136.80	65.63 ± 0.12	119.57	0.17	8433	2
Diameter (mm)	14.22–74.58	47.23 ± 0.08	49.42	0.15	8433	2
Length/width ratio	1.13–3.76	1.44 ± 0.00	0.04	0.14	8433	2

*Note*: Eye depth: low = deep, high = shallow; Flesh color: low = white, high = orange; Skin finish: low = rough, high = smooth; Senescence: low = early, high = late.

The collection of phenotypes includes one ratio trait, length/width ratio, based on two approximately normally distributed traits (length and diameter) (Díaz‐Francés & Rubio, 2013), which was modeled using a normal distribution. Also, for one count trait, tubers/plant, a normal approximation was used to approximate the Poisson distribution due to the high observed lambda parameter (estimated from the mean: λ≅16.2) (Bland, 2000). Additionally, four categorical traits based on either 1–6 or 1–9 manual grading scales (eye depth and skin finish of 1–6 and flesh color and senescence of 1–9, respectively) were also approximated using a normal rather than an ordinal distribution due to the high number of observations. Though some trait distributions were modeled with rather coarse approximations (Figure S6), the collection of traits represents a realistic, diverse set of phenotype distributions in applied breeding programs.

### 3.2 Fertility and the effect of cytoplasm type on agronomic traits

While potatoes are hermaphroditic, in principle allowing for bidirectional crosses with each parent, a widespread lack of fertility in several lines is a signature problem in tetraploid potato breeding, and both male and female sterility are issues (Bethke & Jansky, 2021). Infecundity results from myriad causes, most commonly male sterility, for example, due to cytoplasmic male sterility conferred by cytoplasm types W/γ (Lössl et al., 2000) and D (Sanetomo & Gebhardt, 2015) or meiotic irregularities. Female sterility is an issue to a lesser extent (Bethke & Jansky, 2021). Of the 18 MASPOT parents, five were male sterile, and none were completely female sterile. However, a vast portion (∼50%) of specific crosses were infertile, produced degenerate plants, or failed to generate more than three tubers per plant, which is a practical prerequisite for propagation of the model population. The two W/γ cytoplasm‐type cultivars, Kuras and 07‐LJE‐1 were, expectedly, male sterile, but interestingly, none of the five D‐type parent lines were completely male sterile, despite reports of cytoplasm type D conferring functional male sterility (Hosaka & Sanetomo, 2012; Sanetomo & Gebhardt, 2015). This discrepancy has previously been seen empirically (Sanetomo & Gebhardt, 2015), but the current results constitute a systematic validation of the rule. Indeed, Santayana et al. (2022) showed that combining measures of flowering degree, pollen quantity and viability, and floral abnormalities, rather than solely regarding pollen viability, as is the standard practice, yields a more accurate profile of male fertility. They found that the D cytoplasm genome type performed comparatively better than types T/β and W/γ, with type T/β only being superior in quantities of berries and seeds per berry. This is in accordance with results from our crossing scheme of successful offspring generation in the male cross direction.

The differences between the three cytoplasm types included in the collection of maternal germplasms on trait phenotypes were analyzed by incorporating cytoplasm type as a fixed effect in the prediction models. There could be some confounding between the effect of cytoplasm type and the line effect; however, as the additive and nonadditive genetic contributions are accounted for in the model and the population only includes two reciprocal crosses between parents 04‐GIV‐03 × Rywal and 04‐GIV‐03 × 96‐BYM‐8 (Figure 1), we expect the bias to be minimal. Unfortunately, it was not possible to estimate the amount of variation due to interaction between the nuclear and cytoplasmic genomes due to insufficient statistical power.

Significant differences were found for dry matter content, skin finish, and senescence, while other traits were not significantly influenced by cytoplasm type (Table 2). Cytoplasmic gene effects contributed to a 1.45 and 2.24 percentage point increase of dry matter content in W/γ cytoplasm type clones compared to D and T/β types, respectively, corresponding to 7.16 and 11.05% dry matter content reductions in those cytoplasm types relative to the population mean. Skin finish is increased by ∼0.3 points (i.e., smoother tubers) on the 1–6 scale in D and T/β relative to W/γ (10.08%–10.88% change relative to the mean). Only the Chilean *S. tuberosum*‐derived T/β cytoplasm type (Sanetomo & Gebhardt, 2015) is associated with later senescence (6.60% relative to the mean) compared to the *Solanum demissum* D‐type and wild species W/γ‐type; however, the 0.4‐point effect on the 1–9 scale trait is associated with a large standard error. The W/γ cytoplasm type has previously been correlated with increased tuber starch content (Lössl et al., 2000) as well as yield, late maturity, and more round tubers (Sanetomo & Gebhardt, 2015). Notably, W/γ shows significantly earlier senescence compared to type T/β, in opposition to previous results (Sanetomo & Gebhardt, 2015). However, due to considerable variation of the senescence phenotypes based on manual grading, conclusions relating to senescence are subject to uncertainty. The previously identified association between cytoplasm type and the yield and tuber shape traits could not be reproduced for either of the tuber yield (total yield, tubers/plant) or tuber size (length, diameter, length/width ratio) traits analyzed here. Previous results were based on a population of established cultivars and breeding clones, which is a potential reason why the W/γ cytoplasm type associations that were not reproducible in this study could be due to the frequency of the cytoplasm types, as having only 18 mothers in the diallel cross limits the power of cytoplasm type effect detection, which can also be seen from the substantial standard errors of some of the fixed effect estimates (Table 2).

**TABLE 2:** Estimated contrasts between cytoplasm types on phenotype (± standard error) from the PBLUP_pop_ model.

	Cytoplasm type				
	T/β (frequency 0.56)		D (frequency 0.25)		W/γ (frequency 0.19)
Trait	Fixed effect	% Diff. rel. to mean	Fixed effect	% Diff. rel. to mean	Fixed effect
Dry matter content	−2.24 ± 0.62 (*)	11.05	−1.45 ± 0.64 (*)	7.16	0
Yield	6.48 ± 29.42	1.05	−1.88 ± 31.14	0.31	0
Eye depth	0.10 ± 0.24	3.56	−0.26 ± 0.29	9.85	0
Flesh color	0.54 ± 0.39	10.42	0.55 ± 0.39	10.60	0
Skin finish	0.29 ± 0.12 (*)	10.88	0.27 ± 0.12 (*)	10.08	0
Senescence	0.40 ± 0.18 (*)	6.60	0.16 ± 0.19	2.61	0
Tubers/plant	−0.68 ± 1.12	4.21	0.25 ± 1.17	1.54	0
Length	1.37 ± 2.93	2.09	−0.16 ± 2.97	0.25	0
Diameter	−0.57 ± 1.36	1.21	−0.96 ± 1.41	2.03	0
Length/width ratio	0.06 ± 0.05	4.36	0.03 ± 0.05	1.81	0

*Note*: The contrasts are differences relative to the W/γ cytoplasm type. The percentage change relative to the population mean phenotype is shown.

Abbreviations: Diff rel. to mean, difference relative to mean; PBLUP_pop_, pedigree BLUP on the full MASPOT population.

*95% confidence intervals, not including 0.

Despite the desirable effects of the W/γ on the dry matter content phenotype, increased use of W/γ parental lines in breeding programs would increase male sterility levels, thereby constraining future breeding strategies (Provan et al., 1999; Sanetomo & Gebhardt, 2015). In contrast, purging the breeding pool of the table‐potato market segment of roughness‐promoting W/γ cytoplasm types could contribute to improvement in skin smoothness for the European consumer segment, with this skin texture preference (Pandey et al., 2022). The cytoplasm genomic effects on dry matter content and skin finish could be correlated, as a negative additive genetic correlation between the traits indicates that the genomic variation underpinning the traits is pleiotropic, with increasing dry matter contents being associated with rougher‐skinned tubers (see later results).

Due to the incompleteness of the diallel cross, nonadditive contributions inherent to maternal and paternal crossing direction could not be estimated. Gamete‐specific effects could, however, have illuminated whether and how cross‐direction should be prioritized in breeding. The cytoplasm genomic effects indicate a significant maternally associated effect on some traits.

### 3.3 Additive genetic effects and specific combining abilities

Significant additive genetic variance and variance due to SCAs were found for all traits (Table 3). The breeding value of a clone (=2 GCA) includes at most one‐third of the digenic dominance (Gallais, 2003). In this study, we estimate the additive variance based on all additive genetic relationships. Hence, this effect is expected to be reduced, and the contribution of the digenic dominance is expected to be insignificant. As we are accounting for all other relations in addition to half‐siblings, the digenic dominance can, in practice, contribute a maximum of one‐ninth of the digenic dominance variance to the GCA variance, which will be smaller for the employed dataset.

**TABLE 3:** The additive genetic variance ($\sigma_{a}^{2}$) ± standard error (SE), family effect ($\sigma_{f}^{2}$) ± SE, line effects ($\sigma_{l}^{2}$) ± SE, the percentages of phenotypic variance explained by each (VE [%]) for each component, and the SCA/GCA ratio of all phenotypes (*p < 0.05) calculated by PBLUP_pop_.

Phenotype	$\sigma_{a}^{2}\pm \mathrm{SE}$	VE (%) (h^2^)	$\sigma_{f}^{2}\pm \mathrm{SE}$	VE (%)	$\sigma_{l}^{2}\pm \mathrm{SE}$	VE (%)	SCA/GCA
Dry matter content^a^	2.42 ± 0.19*	29.83	0.38 ± 0.08*	4.67	4.61 ± 0.11*	56.93	0.63
Yield	4712.16 ± 2076.41*	12.64	1918.28 ± 380.78*	5.15	15,473.31 ± 1208.25*	41.51	1.63
Eye depth	0.08 ± 0.06	13.26	0.05 ± 0.02*	7.95	–	–	2.40
Flesh color	1.01 ± 0.37*	49.11	0.06 ± 0.01*	2.94	0.37 ± 0.19	17.83	0.24
Skin finish	0.09 ± 0.03*	15.09	0.01 ± 0.004*	2.58	0.18 ± 0.02	31.22	0.68
Senescence	0.19 ± 0.08*	9.12	0.06 ± 0.01*	2.76	1.12 ± 0.05*	54.66	1.22
Tubers/plant	7.47 ± 3.03*	22.00	1.94 ± 0.37*	5.71	14.21 ± 1.61*	41.85	1.04
Length	58.41 ± 21.78*	54.07	2.47 ± 0.60*	2.28	21.09 ± 10.99	19.52	0.17
Diameter	11.12 ± 4.56*	23.10	2.64 ± 0.51*	5.49	22.24 ± 2.40*	46.22	0.95
Length/width ratio	0.013 ± 0.005*	33.58	0.003 ± 0.0005*	7.50	0.016 ± 0.003*	41.90	0.89

*Note*: For the additive variance explained, the VE was computed as the product of the mean diagonal elements of the covariance matrix (1.001) and the $\sigma_{a}^{2}$ to account for the scaling of the covariance matrix. The percentage of explained phenotypic variance corresponds to the plot narrow‐sense heritability (*h* ^2^).

Abbreviations: GCA, general combining ability; PBLUP_pop_, pedigree BLUP on the full MASPOT population; SCA, specific combining ability.

^a^ Line effect nested within year of test.

The proportion of total genetic variation attributable to additive genetic effects and SCA effects varied across traits, and the predominance of additive versus nonadditive effects was trait‐dependent. Additionally, line effects were considerable for most traits, explaining 18%–57% of the total phenotypic variance. The line effects describe the variance associated with multiple observations of each MASPOT clone. It captures, for example, genetic interactions not captured by the additive and family‐based nonadditive components, as well as line‐specific nonadditive genetic variance. Like the family‐based SCA, the line‐specific nonadditive contributions are non‐transmissible over sexual generations due to recombination and the independent assortment of chromosomes. Particularly, the traits of dry matter content and senescence seem to be associated with strong line‐specific effects of 57% and 55%, respectively. However, a proportion of this variation captured in the line effect is likely also related to the extreme dry matter recordings of a few clones in 2013; that is, some experimental variability is captured in this component. Similarly, the manually graded senescence phenotypes are also prone to inconsistency in the application of the grading scale between graders (Danespo A/S, personal communication, 2024). Overall, the low number of replicates hampers the rigorous estimation of the line effect for individual clones.

**FIGURE 3:**
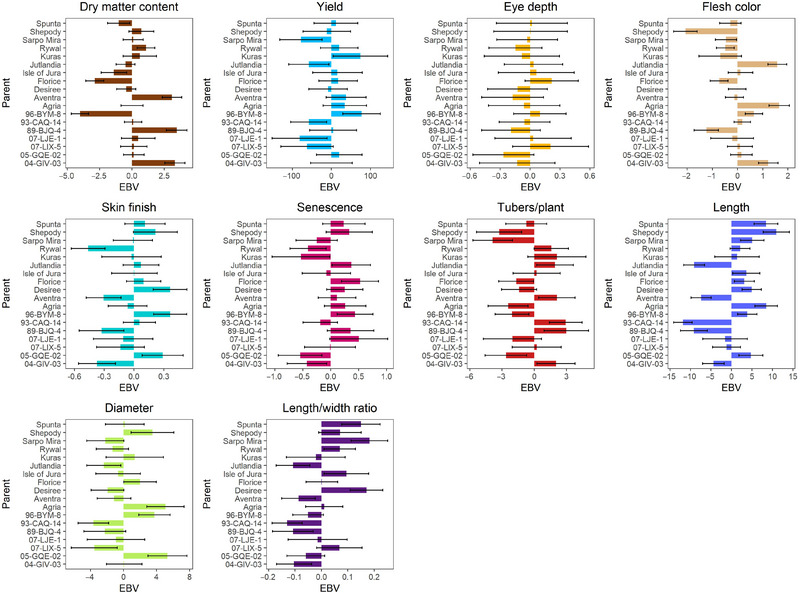
Additive genetic breeding values of MASPOT parents in the unit of the respective phenotypes (with 95% confidence intervals shown as black whiskers) based on single‐step GBLUP (ssGBLUP). EBV, estimated breeding value; GBLUP, best linear unbiased prediction.

High‐heritability traits, such as tuber length and flesh color (Table 3), were found. Interestingly, dry matter content, a known high‐heritability trait (Ortiz et al., 2023), has a narrow‐sense design heritability in the low range (0.31). The narrow‐sense heritability estimated from the regression between the mean corrected phenotype and the mid‐parent phenotype results in a 0.88 ± 0.02 dry matter heritability, consistent with the high heritability expectation for dry matter content. This deflation of the genomic heritability compared to the regression‐based heritability for dry matter content has been encountered in prior studies on the MASPOT panel using standard additive GBLUP (Aalborg et al., 2024; Sverrisdóttir et al., 2017), indicating that some genetic variance is not captured in the additive variance component. However, the specific reason remains obscure. Based on our models, a great portion of the phenotypic variance is captured in the nested line effect, constituting 57% of the total phenotypic variance. This is the highest recorded line effect. While the reason for the heritability‐related discrepancy is obscure, prior genomic prediction models still performed well relative to the regression‐based narrow‐sense heritability and presented with high prediction correlation coefficients (Aalborg et al., 2024; Sverrisdóttir et al., 2017), indicating that EBVs still correlate well with observed performance—the same is also observed in this study (Figure 3). Furthermore, the obtained prediction correlation coefficient of 0.62 using PBLUP_pop_ exceeds the variance explained of the clone means of 0.56 (determined as the square root of the observed design heritability of 0.31). Overall, the deflated additive genetic variance compared to expectations does not seem to affect the EBVs. Dry matter content still presented with greater additive variance (30%) than yield (13%), a twofold difference also found in a previous diallel study (Bradshaw et al., 2000). However, other results of a high specific gravity‐selected population of breeding clones indicated that additive genetic variance of yield exceeded that of specific gravity (closely correlated with dry matter content) (Endelman et al., 2018), highlighting the population dependence of SCA/GCA estimates (Bradshaw, 2022).

Senescence is influenced by a major quantitative trait locus (QTL), as alleles of the chromosome 5 *StCDF1* gene encoding truncated protein variants result in early maturity by affecting mediation between the circadian clock and the tuberization signal (Kloosterman et al., 2013). Estimated additive genetic variance and SCA genetic variance only constituted <15% of the total phenotypic variance. This, despite previous additive models on the genotyped MASPOT panel aptly capturing the *StCDF1* QTL in genome‐wide association studies (Aalborg et al., 2024). Fifty‐five percent of the total variation is attributable to line effects. This possibly reflects the phenotyping method being subject to some inconsistency in scoring, affecting trait data quality. However, the results indicate that all replicates are affected. Eye depth, another major QTL trait, performs similarly, with only 21% variation captured in variance due to additive genetic effects and SCA, and had the highest recorded SCA/GCA ratio of 2.40, comparatively greater than the 1.63 ratio of the complex, polygenic trait yield. Moreover, the additive genetic variance of eye depth is not statistically significant. The known major eye depth QTL, *Eyd*, is located on chromosome 10 in close linkage with the tuber shape *R*_0_ QTL (4 cM), deep eyes being dominant (X. Q. Li et al., 2005; Śliwka et al., 2008). Skin finish shows an additive component (15%), but a sizable proportion of the total variation was captured in the residuals (>50%). Generally, the manually graded phenotypes assessed for the population, apart from flesh color, present substantial line effects and variation not separable from the residuals using the models defined.

Our results indicate that several agronomic traits are mainly governed by additive genetic variance based on SCA/GCA ratios and show good potential for genetic change in the population by breeding. However, we estimate substantial line effects that will likely also include some dominance and epistatic effects not associated with the specific parents, which could indicate that the SCA contribution of some traits is underestimated. Traits relevant to total production quantities, that is, yield, tuber count, and senescence (as a longer growth period is generally associated with increased output), showed higher SCA variance than GCA variance (additive parental contributions), suggesting that transmission of these traits is most influenced by nonadditive effects. However, based on the total additive genetic variance, rather than the GCA variance, all traits were characterized by higher additive genetic variance compared to nonadditive genetic variance, with reservation for possible additional nonadditive variance captured in the line effect. As potato breeding is based on the crossing of selected parent combinations within the breeding population, the total additive genetic variance in the breeding population is important, as it affects the possible genetic gain attainable in progeny generations.

The vegetative propagation of clones that facilitates infinite lifespan entails that the clone is fixed after the first tuber is recovered from sowing the seed. Therefore, the predictions can be used for two purposes: (1) selection of new clones directly for the markets and (2) selection of parents, which can be used for further breeding with very intense selection if we can predict breeding values much earlier in the potato breeding programs. The parent additive genetic value (EBV = 2 GCA) should mainly be used for parental selection, even though some dominance effects are transmittable to progeny (Endelman et al., 2018), and total genetic value can be used for direct selection of new market clones. The total genetic value of a clone will be the sum of the additive and nonadditive genetic effects. Our results regarding the predictions of breeding values of the MASPOT parents across 10 agronomic traits reveal that the additive transmission potential varies greatly across cultivars, both in terms of the number and nature of traits that are effectively transmitted by each parent as well as the transmission ability of each parent (Figure 3, Figure S7). In addition, variation is also considerable in this experiment because the population is defined by sampling parents from different market segments.

While only some traits were influenced most by nonadditive genetic effects based on SCA/GCA ratio, all traits relied on significant nonadditive contributions to the ultimate phenotypic profile. Nonadditive effects are not effectively transmitted to future generations due to recombination and independent assortment of chromosomes. The SCAs of each family (Figures S8–S17) show that only a small subset of families successfully transmits this (ranging between 1 and 12 out of 119 families, depending on the trait). Profiling of SCA is hence a valuable tool in breeding for characterizing cultivar potential. While commercial selection of cultivars for market segments relies on total genetic effects as the vegetative propagation of commercial clones allows a line with an attractive total genetic value to have indefinite life, breeding selection should rely solely on the EBV for parent selection. This distinction is vital, as erroneously attributing trait genetic variation to additive genetic effects rather than SCA results in a notable overestimation of parent potential, and ignoring nonadditive genetic effects might also bias the estimates of additive effects. Therefore, it is highly important to separate the additive and nonadditive genetic effects. As SCA is not accurately proxied by any direct measure, this profiling constitutes an important breeding tool, despite only applying to existing crosses. Testing for market development (clone total genetic value) would be based on additive genetic contribution and SCA effects and would also allow the identification of clones with positive deviations in the genetic part of the line effect, which could contribute to improved total genetic value for a trait relative to the market segment breeding goal. While accurate estimation of total genetic value is important for market clone selection, the overall trait performance of a clone should not be used in parent selection. This should be based on predicted breeding value (additive genetic contribution). A case example of this is the dry matter transmission ability of the starch segment cultivar Kuras. In Denmark, it has historically been a highly popular starch cultivar and consequently has been used in multiple crosses in breeding programs to promote starch content in the offspring. However, our results indicate that the superior starch content in Kuras is not rooted in additive effects but rather nonadditive effects. This is reflected in the low 0.05% dry matter content breeding value of Kuras (prediction error variance [PEV] of 1.31 of the PBLUP_pop_ model for dry matter content). Based on our results, Kuras does not have merit as a dry matter content‐transmitting parent, compared to, for example, Aventra (T/β) with an EBV of 3.44% (PEV of 0.31), besides the increased dry matter content conferred by the undesirable W/γ cytoplasm type. This hypothesis can be validated using historic phenotyping data of, for example, Kuras progeny and Aventra progeny to evaluate dry matter content genetic gain in either parental lineage.

### 3.4 The value of using genomic information in genomic prediction

Generating prediction models trained on phenotypic data alone allows for their implementation in other cultivars in the breeding program based on historical data but requires full phenotyping of each generation before selection. Using pedigree and/or genomic information and subsequent prediction allows for early selection without phenotyping, except for a subset of the breeding pool for model retraining, allowing improved genetic gain in a reduced number of breeding cycles (Lenaerts et al., 2019; Naeem et al., 2021; Ortiz, 2020). We wished to estimate the value of including genotypic data compared to pedigree data on prediction model performance.

**FIGURE 4:**
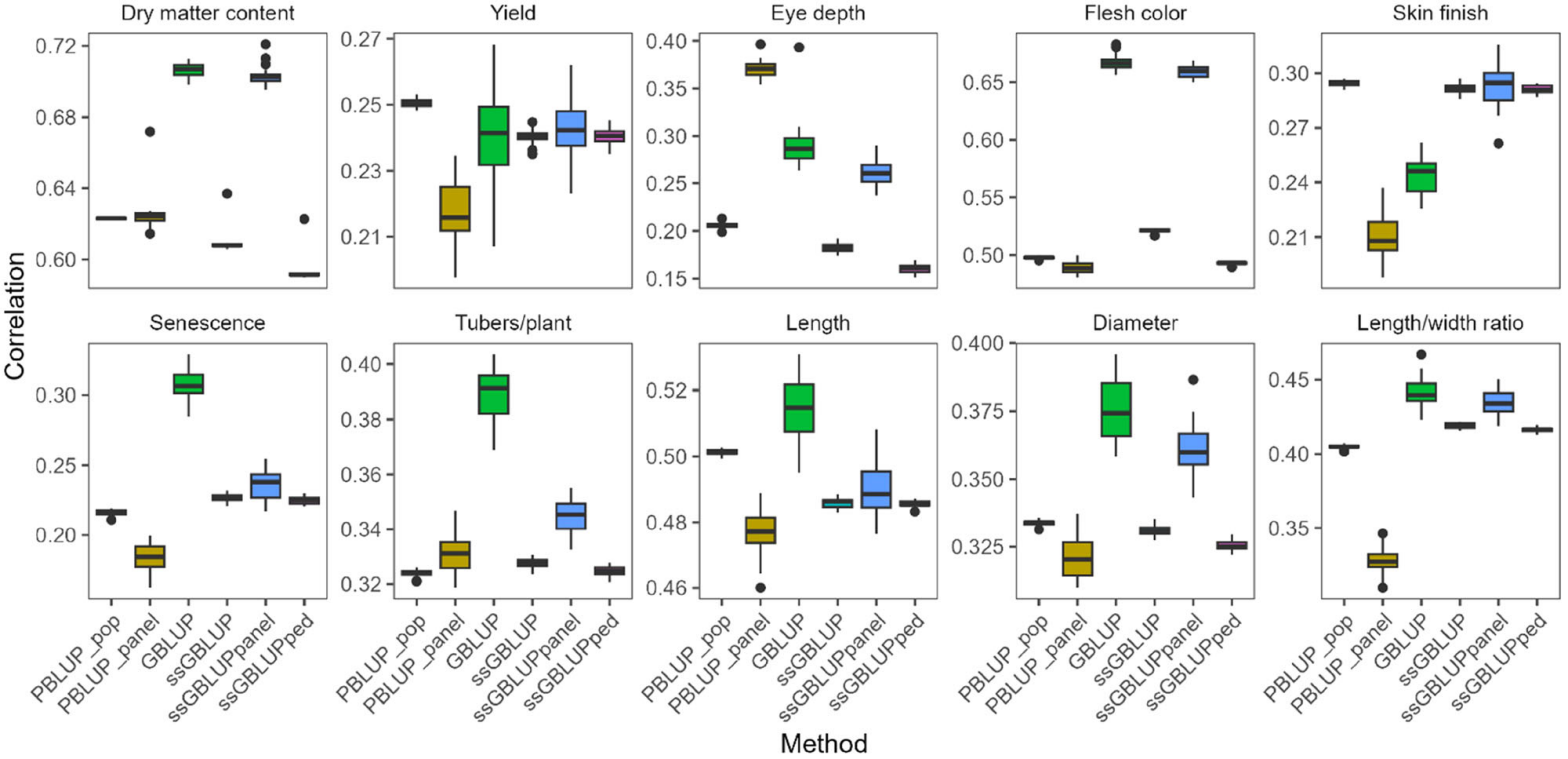
Boxplots of Pearson prediction correlation coefficients [*r*($\bar{y_{c}}$,*â_R_*)] between mean corrected phenotypes ($\bar{y_{c}}$) and estimated breeding values (*â_R_*) for 30 repeats of random eightfold cross‐validation for the four screened models: pedigree BLUP for the full MASPOT population (PBLUP_pop_), pedigree BLUP for the MASPOT panel (PBLUP_panel_), genomic BLUP (GBLUP), and single‐step GBLUP (ssGBLUP). Correlation coefficients of the ssGBLUP were also calculated based on only the genotyped clones (panel) and only the non‐genotyped clones (ped) to illustrate the contribution of genomic information to prediction power. BLUP, best linear unbiased prediction.

All models were validated by 30 repeats of random eightfold cross‐validation, and their performances were evaluated (Figure 4, Table 4, Figure S18, Tables S2–S4). As prediction correlation varied by trait, the value of genomic information for improving model performance also varied. In general, increasing the population size by more than a factor of six from PBLUP_panel_ to PBLUP_pop_ improved the accuracy of PBLUP and, for some traits, significantly improved prediction, for example, for yield, skin finish, senescence, length, and length/width ratio. Including genotypes of the MASPOT panel, a 15% subset of the full population, in an ssGBLUP model resulted in minor variations in correlation coefficients compared to PBLUP_pop_, indicating that pedigree information governs performance at this proportion of genotyped clones. Comparing the performance based on the genotyped versus the non‐genotyped clones in ssGBLUP alludes to the value of adding genotypes. The genotyped clones are associated with greater variation in correlation coefficients between repeats, likely due to fewer individuals in the genotyped panel, but for dry matter content, eye depth, flesh color, tubers/plant, diameter, and length/width ratio, adding genotypes notably improves prediction correlation coefficients. In comparison, using standard additive GBLUP without pedigree information, only on the genotyped MASPOT panel, gave superior models for some traits, particularly for the high heritability trait flesh color and the expected high heritability trait dry matter content, but also for the low (observed) heritability traits of eye depth, senescence, and diameter—this is unexpected. Genomic information can capture cryptic relationships in a population and is generally most advantageous for low‐heritability traits. Furthermore, in this particular population, the genetic structure is very well‐defined, and the effect of each parent is well predicted due to the large progeny groups. This should allow a good prediction of the true breeding value of the parents. However, our results would indicate that the genomic data contributes additional information, and genotyping a higher proportion of the population might contribute to further improvement of prediction accuracies of these high‐heritability traits. Analyzing ssGBLUP models with a stepwise increase of the proportion of individuals genotyped indeed shows that for dry matter content prediction, correlation coefficients improve with the proportion genotyped, and based on the observed trend, a plateau in performance is not yet reached at the currently applied 15% genotyped (Figure S19). Comparatively, an effect of the proportion genotyped is not observed for yield, which is consistent with the similar performance of the PBLUP_pop_ and GBLUP models for this trait. Incorporating genetic information ostensibly improves prediction of the major QTL traits such as eye depth, senescence, and flesh color by including the genetic variation underpinning the bulk of the trait phenotypic variance. A similar effect is also observed for the tuber size and shape measures.

**TABLE 4:** Mean Pearson prediction correlation coefficient between EBV ($\hat{a_{R}}$) in the test set and the mean corrected phenotype ($\bar{y_{c}}$) of 30 repeats of random eightfold cross‐validation ± standard deviation (SD), dispersion bias, plot narrow‐sense heritability (*h* ^2^), design narrow‐sense heritability ($h_{\bar{y_{c}}}^{2}$), and prediction accuracy based on the ssGBLUP model.

Trait	*r*($\bar{y_{c}}$ *, â_R_*) ± SD	Dispersion ($\bar{y_{c}}$, *â_R_*)	*h* ^2^	$h_{\bar{y_{c}}}^{2}$	Prediction accuracy *r*(*a â_R_*)
Dry matter content	0.61 ± 0.005	1.15	0.29	0.31	1.09
Yield	0.24 ± 0.002	1.35	0.15	0.19	0.56
Eye depth	0.18 ± 0.005	1.43	0.18	0.18	0.43
Flesh color	0.52 ± 0.001	1.07	0.58	0.68	0.63
Skin finish	0.29 ± 0.003	1.11	0.21	0.28	0.55
Senescence	0.23 ± 0.003	1.15	0.16	0.19	0.52
Tubers/plant	0.33 ± 0.002	1.16	0.28	0.33	0.57
Length	0.49 ± 0.002	1.07	0.47	0.54	0.67
Diameter	0.33 ± 0.002	1.18	0.34	0.38	0.53
Length/width ratio	0.42 ± 0.002	1.16	0.45	0.49	0.60

*Note*: The prediction accuracy is the correlation between the true breeding value (a) and the EBV.

Abbreviations: EBV, estimated breeding value; GBLUP, best linear unbiased prediction; ssGBLUP, single‐step GBLUP.

Several traits were poorly predicted using either model, namely yield, skin finish, and the major QTL traits senescence and eye depth, with prediction correlations below or close to 0.3 and well below the within‐population estimation of the variance explained based on the square root of the observed heritability. Yield is generally recognized as a complex, highly polygenic trait (van Eck, 2007), and the observed accuracy is consistent with several previous studies (Aalborg et al., 2024; Ortiz, 2020). The PA of each single‐trait ssGBLUP model (Table 4), that is, the ability to predict the breeding value of future clones before they are phenotyped, shows that generally across most traits, prediction accuracies of over 0.52 are achieved, particularly flesh color and length perform well with prediction accuracies above 0.63. An exception to this is eye depth with a lower accuracy of 0.43. Also, the unexpectedly low heritability of dry matter content compared to the high prediction correlation of 0.61 also results in an unrealistic PA of 1.09. While tuber diameter presents with a PA of 0.53, this reflects a relatively low prediction correlation relative to an intermediate heritability. Tuber diameter likely increases to some extent as a function of tuber length, controlled by the dominant *R*_0_ locus, but these results indicate that the genetic inheritance of tuber width is more complexly governed.

The poor prediction correlation of tuber count might reflect the mortality and tuberization selection applied in the MASPOT diallel cross. Solely disqualifying plants based on mortality and tuberization (three per plant) leaves degenerate plants in the population, where tuber growth might be inhibited by overall plant vitality rather than genetic potential. In this scenario, trait performance instead reflects an overall bottleneck. This could be underlying the low plot heritability of tuber count of 0.28 with the ssGBLUP model. Yield could be expected to be subject to similar effects. This is an important consideration of genomic prediction in breeding programs, where selection increases progressively with breeding cycles, and models should be retrained accordingly, so that early models are trained based on populations that include degenerates, and late models are specialized to higher performing individuals with extensive prior selection.

**FIGURE 5:**
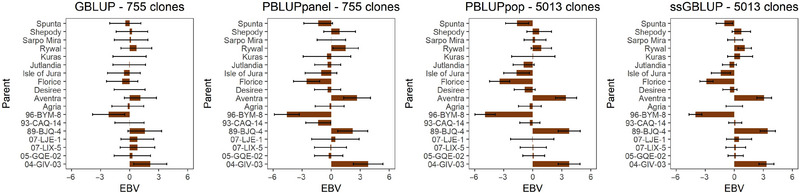
Additive genetic breeding values of MASPOT parents for dry matter content (%) (with 95% confidence interval shown as error bars) predicted with genomic BLUP (GBLUP), PBLUP_panel_, PBLUP_pop_, and single‐step GBLUP (ssGBLUP). BLUP, best linear unbiased prediction; EBV, estimated breeding value; PBLUP, pedigree BLUP.

Reviewing EBVs of dry matter content for the 18 MASPOT parent lines (Figure 5), the predicted estimates are subject to greater uncertainty in the GBLUP and PBLUP_panel_ models with fewer clones due to greater inaccuracy as a result of fewer progeny sampled per parental line. Increasing the population size to ∼5000 clones mildly influenced pedigree EBV values. Also, the inclusion of genomic information in ssGBLUP reduced the standard error of prediction, generating more confident and generally more conservative EBV predictions. These overall trends are observed for all analyzed traits (Figures S20–S28). The more conservative EBV predictions for ssGBLUP are unexpected, as adding genomic information normally amplifies EBV predictions, but the response observed is likely a result of the specific diallel experimental design.

Our results show that including genotype information in ssGBLUP models has the potential to enhance PA compared to traditional PBLUP, but that the proportion of genotyped clones in the population (15%) was too small to influence overall model performance. Increasing the fraction of individuals genotyped is expected to increase ssGBLUP prediction accuracies (Song et al., 2019), and a similar influence of population size is expected for GBLUP, as this would increase the portion of total genetic variance explained by genotype. Accounting for more individual variation by increasing genotyped fraction will likely also generate more accurate, and hypothetically also more conservative, predictions of breeding values when using ssGBLUP. To extrapolate upon this, we argue that genotyping efforts should be aimed at increasing the size of the genotyped panels to improve model performance. Additionally, extending the panel diversity will also improve the predictive ability for future extra‐population clones (Sverrisdóttir et al., 2018). Comparatively, the value of intensifying or maintaining phenotyping efforts beyond what is needed for generating a retraining dataset for genomic prediction models relative to the cost and additional time required before possible selection is doubtful. Based on our results, the additive genetic variance was sufficiently captured with a 755‐clone population in PBLUP, as EBV predictions changed only slightly with a factor of more than a sixfold increase in phenotyped population, indicating that the genetic variance contained in the pedigree of this 18‐parent diallel is widely accounted for with a smaller population. However, for application in a general breeding program, where expanding the population size involves increasing the number of parent combinations, a comparatively larger population will likely also be required to capture the additive genetic variance. By increasing population size, a more precise estimate of the genetic variance can be obtained, and adding genetic information about new families of the diallel crossing design in addition to those already covered in the incomplete diallel would be further advantageous.

In conclusion, the value of adding genotypic data is context dependent, as can be observed from the prediction of EBVs in GBLUP compared to PBLUP and ssGBLUP. The mean additive contribution of a parent to a population of offspring is proxied by the mid‐parent value and contained in the pedigree, whereas genotypes are useful in describing the individual clone's deviation from the mean parental additive genetic value. The differing levels of information carried are apparent from the large uncertainty associated with the GBLUP additive genetic breeding value predictions of the parents compared to the pedigree‐based predictions. This is contrasted by the performance boost of the panel‐wide prediction correlation of some traits using a GBLUP compared to the PBLUP model.

### 3.5 Genetic correlations

**FIGURE 6:**
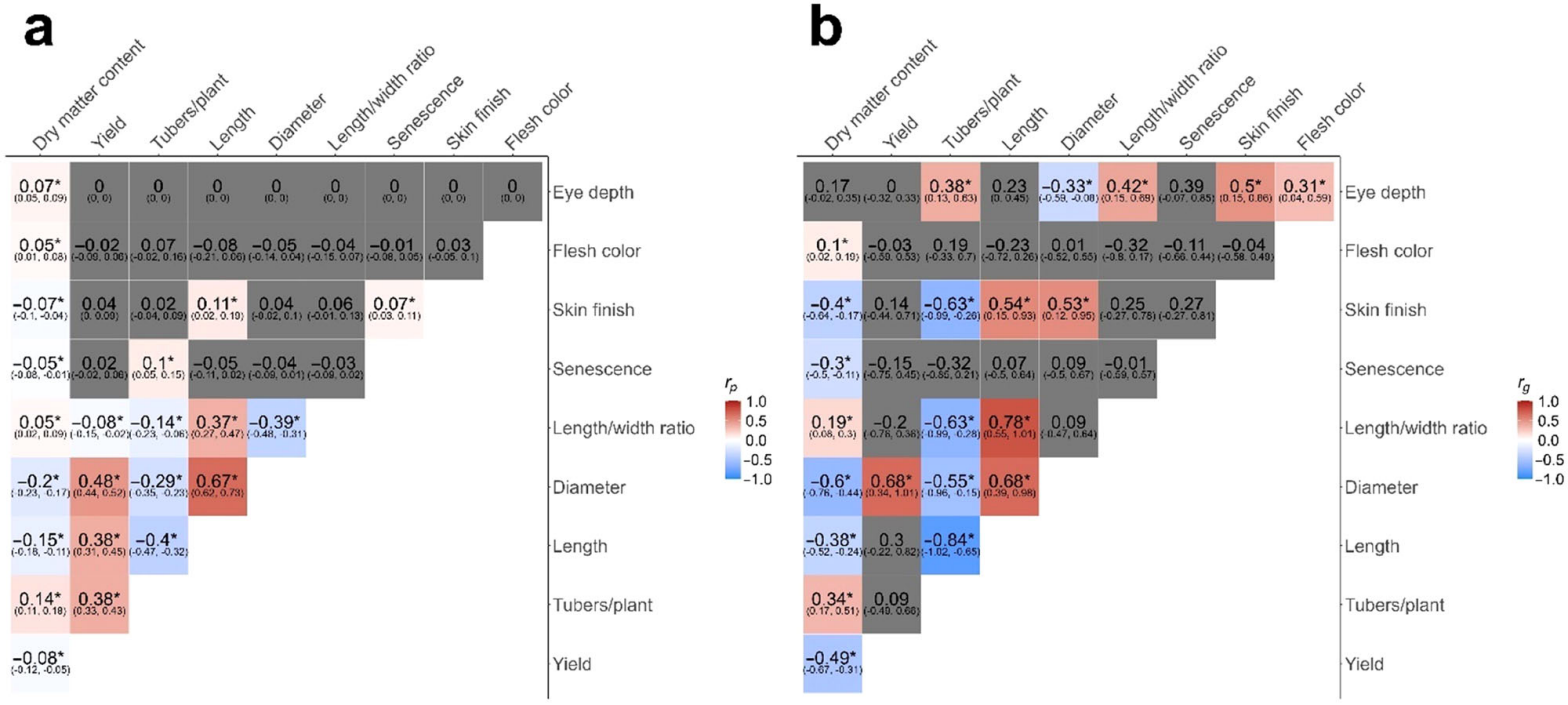
Pairwise (a) phenotypic and (b) additive genetic correlations between traits with 95% confidence intervals based on PBLUP_pop_. (*denotes confidence intervals not crossing 0, and correlations with confidence intervals including 0 are shown in gray). Phenotypic correlations could not be computed for trait combinations including eye depth other than dry matter content, as eye depth was recorded without replicates in 2014, and a residual covariance could not be computed.

The phenotypic and additive genetic correlations between all pairwise trait combinations were evaluated by fitting bivariate PBLUP_pop_ models (Figure 6). Overall, while few trait pairs showed strong phenotypic correlations, the statistically significant genetically correlated traits presented with greater absolute values, but consistently same direction, correlations as compared to the phenotypic. The higher the heritability of the traits involved in the correlation, the more the genetic parts also influence the phenotypic correlation. Dry matter content and eye depth were genetically correlated with most other traits analyzed. The *Eyd* locus for eye depth is genetically linked to the tuber shape *R*_0_ locus, controlled by the *StOFP20* gene (S. Wu, Zhang, et al., 2018), with a distance of only 4 cM; rounder tubers being associated with deeper eyes (X. Q. Li et al., 2005; Śliwka et al., 2008), which was replicated here (0.42). In relation to this, the increased tuber count is also correlated with shallow eyes (0.38). As tuber count is inversely correlated with tuber size measures, both length (−0.84) and diameter (−0.55), this shows a propensity toward a tuberization dichotomy of generating either few, large tubers or a manifold of small tubers to store the assimilated carbon. The shallow eyes of tuber‐rich clones are likely a result of the small tubers (pleiotropy).

Similar results were also found for dry matter, with genetic correlations indicating increased dry matter content in smaller, oblong tubers. Oblong tuber shape has previously been linked to starch content (Urbany et al., 2011), possibly a result of several starch metabolism genes coinhabiting the chromosome 10 South arm along with the *R*_0_ locus (Schreiber et al., 2014). Dry matter content had a strong negative genetic correlation with total yield (−0.49), despite not presenting with a notable phenotypic correlation between the traits (−0.08). Other studies have also found negative genetic correlations of −0.18 and −0.24 between starch content and tuber yield (L. Li et al., 2013; Urbany et al., 2011), which are comparably weaker than the −0.49 reported here. This might reflect the inclusion of both starch and protein content in the joint dry matter content phenotype. As an enhancement of yield entails a reduction in dry matter content, the gained tuber weight likely arises from surged water contents. Genetic variation in the starch metabolism genes could result in disparate enzymatic profiles that catalyze the formation of distinct starch structures in low‐ and high‐starch tubers, where more heavily phosphorylated polymers and/or increased amylose/amylopectin ratio result in granule swelling by inflated water‐binding capacity in the amorphous lamella in low‐starch clones, compared to the starch structures in high‐dry matter content cultivars, where water retention capacity in the tuber storage polymer is inhibited. Indeed, the amylose/amylopectin ratio and crystallinity (negatively correlated with amylose content) are genotype‐dependent and impact starch granule size, with larger granules presenting with higher amylose contents (Schirmer et al., 2013; Tong et al., 2023). Dry matter content is also negatively genetically correlated with tuber size measures (diameter [−0.6] and length [−0.38]) and positively with tuber count (0.34), which could further indicate that low swelling power results in the formation of smaller tubers in higher numbers.

In previous findings, both high yield and dry matter contents have been phenotypically correlated with late maturity, as the reduced vegetation period of early senescing plants compared to late‐maturing cultivars results in reduced yield and dry matter content, muting genetic influences on these traits; that is, the maturity type is determinant of the phenotypic outputs of these traits (Urbany et al., 2011; van Eck, 2007). We did not find any genetic correlations between senescence and yield or dry matter content underpinning this effect. This might, however, be due to the low quality of senescence phenotypes.

Additive genetic correlations relating to tuber shape traits, that is, length, diameter, and length/width ratio, are consonant with expectations based on phenotypic correlations, with length/width ratio being positively correlated with length (0.78), and length also being positively correlated with diameter (0.68). This indicates a genetic control of both radial and apical tuber extension. Smoother skin and yellow flesh color correlated genetically with shallow eye depth (genetic correlations of 0.5 and 0.31, respectively), a genetic feature that is relevant in selection for processing and table potato markets, mentioning that preferences are highly variable across countries. In addition, skin finish is negatively genetically correlated with dry matter content (−0.4). The starch segment is largely unselected for smooth skin finish, and cultivars generally generate rough‐skinned tubers. Hence, this association could be population dependent, having arisen in the Danespo A/S breeding population, which we sampled through the 18 MASPOT parents, rather than being a true genetic correlation. Similarly, long tubers correlate with a smoother skin finish (0.54), a trait combination generally selected for in the French fry processing market (Van Eck et al., 1994). However, these results would also indicate a genetic mode of inheritance linking these traits due to pleiotropic effects; for example, a pleiotropic effect of the *R*_0_ locus, where tuber elongation entails stretching of the skin to form a smoother surface. This effect could, however, also be specific to the assessed population due to two genetically linked loci.

Overall, the weak (0.1) to strong (−0.84) genetic correlations observed between pairwise trait combinations for the MASPOT population indicate that selection for any one trait during breeding will entail the indirect selection of up to several other important traits. Current selection in breeding programs is routinely based on multiple traits concurrently, but selection schemes typically employ an approach of sequential, single‐trait disqualification criteria, which has the obvious disadvantage of diminishing selection indices of later traits. We do not expect bivariate models or multivariate models to be much more accurate predictors for any single trait compared to single‐trait models. However, for breeding purposes, it is important to discern whether genetic optimization of one trait results in indirect selection of other traits due to underlying genetic correlations. Multi‐trait models are hence more suitable to optimize prediction models implemented to support selection (Neyhart et al., 2019; Ortiz et al., 2023), as they better capture correlated genetic features (Song et al., 2019). Therefore, an interesting perspective is to quantify the increased genetic gain of employing multi‐trait models. In principle, the MASPOT panel data would be suitable for such a study, though using an actual breeding population compared to an experimental population will likely produce more accurate estimates of increased genetic gain. Software is also available for the simulation of an autotetraploid potato breeding population that could be used in such a study (Chu & Jensen, 2025).

## 4 CONCLUSIONS

In this study, we explored the transmission ability of a collection of advanced breeding clones for a collection of agronomic traits using an unselected F_1_ training population to explore means of trait genetic improvement through breeding. Expectedly, traits differed in their partitioning of genetic variance between predominantly additive or nonadditive, but all traits possessed significant additive genetic effects that have potential for genetic change in the population through breeding. Significant contributions to some trait phenotypes were found to be conferred by cytoplasmic genome types, indicating that the parent cytoplasmic genome type is an important consideration during crossing designs. Overall, the assessed parental cultivars displayed highly diverse transmission abilities across traits, and a network of pairwise trait genetic correlations indicates that single‐trait selection is associated with substantial indirect trait selection. It could be relevant to explore multi‐trait selection models to account for this co‐selection in the future of potato breeding. The inclusion of GBS information (available for 15% of the experimental population) in addition to phenotypic information in a single‐step approach improved the accuracy of cultivar EBV prediction, but for genomic prediction models, the optimal overall PA was obtained using GBLUP. Our results contribute to the mapping of transmission ability for the specific cultivars analyzed but also highlight the complex genetic inheritance and prediction of the total genetic value of clones in breeding.

## AUTHOR CONTRIBUTIONS

**Trine Aalborg**: Conceptualization; data curation; formal analysis; investigation; methodology; visualization; writing—original draft; writing—review and editing. **Hélène Romé**: Formal analysis; methodology; writing—review and editing. **Christina Ranzau**: Data curation; writing—review and editing. **Merethe Bagge**: Data curation; writing—review and editing. **Just Jensen**: Conceptualization; formal analysis; methodology; supervision; writing—review and editing. **Kåre Lehmann Nielsen**: Conceptualization; funding acquisition; methodology; supervision; writing—review and editing.

## CONFLICT OF INTEREST STATEMENT

Christina Ranzau and Merethe Bagge are employed by Danespo A/S, Denmark. Kåre Lehmann Nielsen is partially employed by KMC Amba, Denmark. Hélène Romé is currently affiliated with SEGES Innovation P/S, Denmark. The remaining authors declare that they have no conflicts of interest.

## Supporting information



Additional supporting information can be found online in the Supplemental Material section at the end of this article. These include supplementary figures (**Supplemental Material S1**) and supplementary tables (**Supplemental Material S2**).

Supplemental Material

## Data Availability

The datasets needed to reproduce the results of this study are available in the Zenodo repositor: https://doi.org/10.5281/zenodo.11916143. These include that marker and pedigree data of the population, as well as recorded phenotypic and metadata.

## References

[tpg270066-bib-0001] Aalborg, T. , & Nielsen, K. L. (2024). To be or not to be tetraploid—The impact of marker ploidy on genomic prediction and GWAS of potato. Frontiers in Plant Science, 15, 1386837. 10.3389/fpls.2024.1386837 39139728 PMC11319270

[tpg270066-bib-0002] Aalborg, T. , Sverrisdóttir, E. , Kristensen, H. T. , & Nielsen, K. L. (2024). The effect of marker types and density on genomic prediction and GWAS of key performance traits in tetraploid potato. Frontiers in Plant Science, 15, 1340189. 10.3389/fpls.2024.1340189 38525152 PMC10957621

[tpg270066-bib-0003] Amadeu, R. R. , Garcia, A. A. F. , Munoz, P. R. , & Ferrão, L. F. V. (2023). AGHmatrix: Genetic relationship matrices in R. Bioinformatics, 39, btad445. 10.1093/BIOINFORMATICS/BTAD445 37471595 PMC10371492

[tpg270066-bib-0004] Bethke, P. C. , & Jansky, S. H. (2021). Genetic and environmental factors contributing to reproductive success and failure in potato. American Journal of Potato Research, 98, 24–41. 10.1007/s12230-020-09810-3

[tpg270066-bib-0005] Bland, M. (2000). An introduction to medical statistics (3rd ed.). Oxford University Press.

[tpg270066-bib-0006] Bourke, P. M. , Voorrips, R. E. , Visser, R. G. F. , & Maliepaard, C. (2015). The double‐reduction landscape in tetraploid potato as revealed by a high‐density linkage map. Genetics, 201, 853–863. 10.1534/GENETICS.115.181008/-/DC1 26377683 PMC4649655

[tpg270066-bib-0007] Bourke, P. M. , Voorrips, R. E. , Visser, R. G. F. , & Maliepaard, C. (2018). Tools for genetic studies in experimental populations of polyploids. Frontiers in Plant Science, 9, Article 513. 10.3389/FPLS.2018.00513 29720992 PMC5915555

[tpg270066-bib-0008] Bradshaw, J. E. (2022). A brief history of the impact of potato genetics on the breeding of tetraploid potato cultivars for tuber propagation. Potato Research, 65, 461–501. 10.1007/s11540-021-09517-w

[tpg270066-bib-0009] Bradshaw, J. E. , Todd, D. , & Wilson, R. N. (2000). Use of tuber progeny tests for genetical studies as part of potato (*Solanum tuberosum* subsp. *tuberosum*) breeding programme. Theoretical and Applied Genetics [Theoretische Und Angewandte Genetik], 100, 772–781. 10.1007/s001220051351 24172936

[tpg270066-bib-0010] Brown, C. R. (1993). Origin and history of the potato. American Potato Journal, 70, 363–373. 10.1007/BF02849117

[tpg270066-bib-0011] Christensen, O. F. , & Lund, M. S. (2010). Genomic prediction when some animals are not genotyped. Genetics Selection Evolution, 42, Article 2. 10.1186/1297-9686-42-2 PMC2834608 20105297

[tpg270066-bib-0012] Chu, T. T. , & Jensen, J. (2025). ADAM‐multi: Software to simulate complex breeding programs for animals and plants with different ploidy levels and generalized genotypic effect models to account for multiple alleles. Frontiers in Genetics, 16, 1513615. 10.3389/FGENE.2025.1513615 39995464 PMC11847855

[tpg270066-bib-0013] Darabad, G. R. , Hassandokht, M. R. , Hassanpanah, D. , & Mousavi, A. (2020). Diallel cross in potato cultivars (*Solanum tuberosum* L.) and evaluation of their progenies under deficit water stress. Acta Agrobotanica, 73, 1–9. 10.5586/AA.7325

[tpg270066-bib-0014] Dean, R. B. , & Dixon, W. J. (1951). Simplified statistics for small numbers of observations. Analytical Chemistry, 23, 636–638. 10.1021/ac60052a025

[tpg270066-bib-0015] Devaux, A. , Goffart, J.‐P. , Petsakos, A. , Kromann, P. , Gatto, M. , Okello, J. , Suarez, V. , & Hareau, G. (2019). Global food security, contributions from sustainable potato agri‐food systems. In H. Campos & O. Ortiz (Eds.), The potato crop: Its agricultural, nutritional and social contribution to humankind (pp. 3–36). Springer International Publishing.

[tpg270066-bib-0016] Devaux, A. , Kromann, P. , & Ortiz, O. (2014). Potatoes for sustainable global food security. Potato Research, 57, 185–199. 10.1007/S11540-014-9265-1

[tpg270066-bib-0017] Díaz‐Francés, E. , & Rubio, F. J. (2013). On the existence of a normal approximation to the distribution of the ratio of two independent normal random variables. Statistical Papers, 54, 309–323. 10.1007/S00362-012-0429-2/METRICS

[tpg270066-bib-0018] Endelman, J. B. , Carley, C. A. S. , Bethke, P. C. , Coombs, J. J. , Clough, M. E. , Da Silva, W. L. , De Jong, W. S. , Douches, D. S. , Frederick, C. M. , Haynes, K. G. , Holm, D. G. , Miller, J. C. , Muñoz, P. R. , Navarro, F. M. , Novy, R. G. , Palta, J. P. , Porter, G. A. , Rak, K. T. , Sathuvalli, V. R. , … Yencho, G. C. (2018). Genetic variance partitioning and genome‐wide prediction with allele dosage information in autotetraploid potato. Genetics, 209, 77–87. 10.1534/GENETICS.118.300685 29514860 PMC5937173

[tpg270066-bib-0019] FAOSTAT . (2024). Crops and livestock products. Food and Agriculture Organization of the United Nations Statistics Division. https://www.fao.org/faostat/en/#data/QCL

[tpg270066-bib-0020] Gallais, A. (2003). Quantitative genetics and breeding methods in autopolyploid plants (1st ed.). INRA.

[tpg270066-bib-0021] Gebhardt, C. (2016). The historical role of species from the Solanaceae plant family in genetic research. Theoretical and Applied Genetics, 129, 2281–2294. 10.1007/s00122-016-2804-1 27744490 PMC5121179

[tpg270066-bib-0022] Gerard, D. (2023). Double reduction estimation and equilibrium tests in natural autopolyploid populations. Biometrics, 79, 2143–2156. 10.1111/biom.13722 35848417

[tpg270066-bib-0023] Gopal, J. (1998). General combining ability and its repeatability in early generations of potato breeding programmes. Potato Research, 41, 21–28. 10.1007/BF02360258

[tpg270066-bib-0024] Griffing, B. (1956). Concept of general and specific combining ability in relation to diallel crossing systems. Australian Journal of Biological Sciences, 9, 463–493. 10.1071/BI9560463

[tpg270066-bib-0025] Gutaker, R. M. , Weiß, C. L. , Ellis, D. , Anglin, N. L. , Knapp, S. , Luis Fernández‐Alonso, J. , Prat, S. , & Burbano, H. A. (2019). The origins and adaptation of European potatoes reconstructed from historical genomes. Nature Ecology & Evolution, 3(7), 1093–1101. 10.1038/s41559-019-0921-3 31235927

[tpg270066-bib-0026] Haase, N. U. (2003). Estimation of dry matter and starch concentration in potatoes by determination of under‐water weight and near infrared spectroscopy. Potato Research, 46, 117–127. 10.1007/BF02736081

[tpg270066-bib-0027] Hardy, O. J. (2016). Population genetics of autopolyploids under a mixed mating model and the estimation of selfing rate. Molecular Ecology Resources, 16, 103–117. 10.1111/1755-0998.12431 25981126

[tpg270066-bib-0028] Hickey, J. M. , Chiurugwi, T. , Mackay, I. , & Powell, W. (2017). Genomic prediction unifies animal and plant breeding programs to form platforms for biological discovery. Nature Genetics, 49, 1297–1303. 10.1038/NG.3920 28854179

[tpg270066-bib-0029] Hosaka, K. , & Sanetomo, R. (2012). Development of a rapid identification method for potato cytoplasm and its use for evaluating Japanese collections. Theoretical and Applied Genetics, 125, 1237–1251. 10.1007/S00122-012-1909-4/FIGURES/4 22696007

[tpg270066-bib-0030] Hutten, R. , & van Berloo, R. (2001). An online potato pedigree database . Wageningen University & Research. http://www.plantbreeding.wur.nl/PotatoPedigree/

[tpg270066-bib-0031] Kloosterman, B. , Abelenda, J. A. , Gomez, M. D. M. C. , Oortwijn, M. , De Boer, J. M. , Kowitwanich, K. , Horvath, B. M. , Van Eck, H. J. , Smaczniak, C. , Prat, S. , Visser, R. G. F. , & Bachem, C. W. B. (2013). Naturally occurring allele diversity allows potato cultivation in northern latitudes. Nature, 495(7440), 246–250. 10.1038/nature11912 23467094

[tpg270066-bib-0032] Kristensen, P. S. , Sarup, P. , Fé, D. , Orabi, J. , Snell, P. , Ripa, L. , Mohlfeld, M. , Chu, T. T. , Herrström, J. , Jahoor, A. , & Jensen, J. (2023). Prediction of additive, epistatic, and dominance effects using models accounting for incomplete inbreeding in parental lines of hybrid rye and sugar beet. Frontiers in Plant Science, 14, 1193433. 10.3389/FPLS.2023.1193433 38162304 PMC10756082

[tpg270066-bib-0033] Legarra, A. , Aguilar, I. , & Misztal, I. (2009). A relationship matrix including full pedigree and genomic information. Journal of Dairy Science, 92, 4656–4663. 10.3168/jds.2009-2061 19700729

[tpg270066-bib-0034] Lenaerts, B. , Collard, B. C. Y. , & Demont, M. (2019). Review: Improving global food security through accelerated plant breeding. Plant Science, 287, 110207. 10.1016/j.plantsci.2019.110207 31481198 PMC6745619

[tpg270066-bib-0035] Li, L. , Tacke, E. , Hofferbert, H.‐R. , Lübeck, J. , Strahwald, J. , Draffehn, A. M. , Walkemeier, B. , & Gebhardt, C. (2013). Validation of candidate gene markers for marker‐assisted selection of potato cultivars with improved tuber quality. Theoretical and Applied Genetics, 126, 1039–1052. 10.1007/S00122-012-2035-Z 23299900 PMC3607734

[tpg270066-bib-0036] Li, X. Q. , De Jong, H. , De Jong, D. M. , & De Jong, W. S. (2005). Inheritance and genetic mapping of tuber eye depth in cultivated diploid potatoes. Theoretical and Applied Genetics, 110, 1068–1073. 10.1007/s00122-005-1927-6 15719211

[tpg270066-bib-0037] Lian, Q. , Zhang, S. , Wu, Z. , Zhang, C. , & Negrão, S. (2024). Assembly and comparative analysis of the mitochondrial genome in diploid potatoes. Plant Cell Reports, 43, Article 49. 10.1007/S00299-024-03326-4 39358565

[tpg270066-bib-0038] Lössl, A. , Götz, M. , Braun, A. , & Wenzel, G. (2000). Molecular markers for cytoplasm in potato: Male sterility and contribution of different plastid‐mitochondrial configurations to starch production. Euphytica, 116, 221–230. 10.1023/A:1004039320227

[tpg270066-bib-0039] Luan, T. , Woolliams, J. A. , Lien, S. , Kent, M. , Svendsen, M. , & Meuwissen, T. H. E. (2009). The accuracy of genomic selection in Norwegian red cattle assessed by cross‐validation. Genetics, 183, 1119–1126. 10.1534/GENETICS.109.107391 19704013 PMC2778964

[tpg270066-bib-0080] Madsen, P. , Jensen, J. , Labouriau, R. , Christensen, O. F. , & Sahana, G. (2014). DMU ‐ A package for analyzing multivariate mixed models in quantitative genetics and genomics. In Proceedings, 10th World Congress of Genetics Applied to Livestock Production. American Society of Animal Science.

[tpg270066-bib-0041] Martini, J. W. R. , Schrauf, M. F. , Garcia‐Baccino, C. A. , Pimentel, E. C. G. , Munilla, S. , Rogberg‐Muñoz, A. , Cantet, R. J. C. , Reimer, C. , Gao, N. , Wimmer, V. , & Simianer, H. (2018). The effect of the H^−1^ scaling factors *τ* and *ω* on the structure of H in the single‐step procedure. Genetics Selection Evolution, 50, Article 16. 10.1186/S12711-018-0386-X/FIGURES/2 PMC5899415 29653506

[tpg270066-bib-0042] Meuwissen, T. H. E. , Hayes, B. J. , & Goddard, M. E. (2001). Prediction of total genetic value using genome‐wide dense marker maps. Genetics, 157, 1819–1829. 10.1093/GENETICS/157.4.1819 11290733 PMC1461589

[tpg270066-bib-0043] Naeem, M. , Demirel, U. , Yousaf, M. F. , Caliskan, S. , & Caliskan, M. E. (2021). Overview on domestication, breeding, genetic gain and improvement of tuber quality traits of potato using fast forwarding technique (GWAS): A review. Plant Breeding, 140, 519–542. 10.1111/PBR.12927

[tpg270066-bib-0044] Neyhart, J. L. , Lorenz, A. J. , & Smith, K. P. (2019). Multi‐trait improvement by predicting genetic correlations in breeding crosses. G3: Genes, Genomes, Genetics, 9, 3153–3165. 10.1534/g3.119.400406 31358561 PMC6778794

[tpg270066-bib-0045] Ortiz, R. (2020). Genomic‐led potato breeding for increasing genetic gains: Achievements and outlook. Crop Breeding, Genetics and Genomics, 2, e200010. 10.20900/CBGG20200010

[tpg270066-bib-0046] Ortiz, R. , Iwanaga, M. , & Mendoza, H. A. (1988). Combining ability and parental effects in 4x‐2x crosses for potato breeding. Potato Research, 31, 643–650. 10.1007/BF02361857

[tpg270066-bib-0047] Ortiz, R. , Reslow, F. , Montesinos‐López, A. , Huicho, J. , Pérez‐Rodríguez, P. , Montesinos‐López, O. A. , & Crossa, J. (2023). Partial least squares enhance multi‐trait genomic prediction of potato cultivars in new environments. Scientific Reports, 13(1), Article 9947. 10.1038/s41598-023-37169-y 37336933 PMC10279678

[tpg270066-bib-0048] Pandey, J. , Scheuring, D. C. , Koym, J. W. , Endelman, J. B. , & Vales, M. I. (2023). Genomic selection and genome‐wide association studies in tetraploid chipping potatoes. Plant Genome, 16, e20297. 10.1002/tpg2.20297 36651146 PMC12807239

[tpg270066-bib-0049] Pandey, J. , Scheuring, D. C. , Koym, J. W. , & Vales, M. I. (2022). Genomic regions associated with tuber traits in tetraploid potatoes and identification of superior clones for breeding purposes. Frontiers in Plant Science, 13, 952263. 10.3389/fpls.2022.952263 35937326 PMC9354404

[tpg270066-bib-0050] Parisod, C. , Holderegger, R. , & Brochmann, C. (2010). Evolutionary consequences of autopolyploidy. The New Phytologist, 186, 5–17. 10.1111/j.1469-8137.2009.03142.x 20070540

[tpg270066-bib-0051] Plaisted, R. L. , Sanford, L. , Federer, W. T. , Kehr, A. E. , & Peterson, L. C. (1962). Specific and general combining ability specific and general combining ability for yield in potatoes. American Potato Journal, 39, 185–197. 10.1007/BF02871402

[tpg270066-bib-0052] Posit Team . (2023). RStudio: Integrated development environment for R .

[tpg270066-bib-0053] Provan, J. , Powell, W. , Dewar, H. , Bryan, G. , Machray, G. C. , & Waugh, R. (1999). An extreme cytoplasmic bottleneck in the modern European cultivated potato (*Solanum tuberosum*) is not reflected in decreased levels of nuclear diversity. Proceedings of the Royal Society of London. Series B: Biological Sciences, 266, 633–639. 10.1098/rspb.1999.0683

[tpg270066-bib-0054] R Core Team . (2023). R: A language and environment for statistical computing .

[tpg270066-bib-0055] Rorabacher, D. B. (1991). Statistical treatment for rejection of deviant values: Critical value of Dixon's “Q” parameter and related subrange ratios at the 95% confidence level. Analytical Chemistry, 63, 139–146.

[tpg270066-bib-0056] Ruiz De Arcaute, R. , Carrasco, A. , Ortega, F. , Rodriguez‐Quijano, M. , & Carrillo, J. M. (2022). Evaluation of genetic resources in a potato breeding program for chip quality. Agronomy, 12(5), 1142. 10.3390/agronomy12051142

[tpg270066-bib-0057] Sanetomo, R. , & Gebhardt, C. (2015). Cytoplasmic genome types of European potatoes and their effects on complex agronomic traits. BMC Plant Biology, 15, Article 162. 10.1186/S12870-015-0545-Y 26112802 PMC4480903

[tpg270066-bib-0058] Santayana, M. , Aponte, M. , Kante, M. , Eyzaguirre, R. , Gastelo, M. , & Lindqvist‐Kreuze, H. (2022). Cytoplasmic male sterility incidence in potato breeding populations with late blight resistance and identification of breeding lines with a potential fertility restorer mechanism. Plants, 11(22), 3093. 10.3390/PLANTS11223093 36432822 PMC9696232

[tpg270066-bib-0059] Schirmer, M. , Höchstötter, A. , Jekle, M. , Arendt, E. , & Becker, T. (2013). Physicochemical and morphological characterization of different starches with variable amylose/amylopectin ratio. Food Hydrocolloids, 32, 52–63. 10.1016/j.foodhyd.2012.11.032

[tpg270066-bib-0079] Schreiber, L. , Nader‐Nieto, A. C. , Schönhals, E. M. , Walkemeier, B. , & Gebhardt, C. (2014). SNPs in genes functional in starch‐sugar interconversion associate with natural variation of tuber starch and sugar content of potato (*Solanum tuberosum* L.). G3 Genes|Genomes|Genetics, 4(10), 1797–1811. 10.1534/g3.114.012377 25081979 PMC4199688

[tpg270066-bib-0060] Slater, A. T. , Cogan, N. O. I. , Forster, J. W. , Hayes, B. J. , & Daetwyler, H. D. (2016). Improving genetic gain with genomic selection in autotetraploid potato. Plant Genome, 9, plantgenome2016.02.0021. 10.3835/PLANTGENOME2016.02.0021 27902807

[tpg270066-bib-0061] Śliwka, J. , Wasilewicz‐Flis, I. , Jakuczun, H. , & Gebhardt, C. (2008). Tagging quantitative trait loci for dormancy, tuber shape, regularity of tuber shape, eye depth and flesh colour in diploid potato originated from six *Solanum* species. Plant Breeding, 127, 49–55. 10.1111/j.1439-0523.2008.01420.x

[tpg270066-bib-0062] Song, H. , Zhang, J. , Zhang, Q. , & Ding, X. (2019). Using different single‐step strategies to improve the efficiency of genomic prediction on body measurement traits in pig. Frontiers in Genetics, 10. 10.3389/fgene.2018.00730 PMC6340005 30693018

[tpg270066-bib-0063] Spooner, D. M. , Mclean, K. , Ramsay, G. , Waugh, R. , & Bryan, G. J. (2005). A single domestication for potato based on multilocus amplified fragment length polymorphism genotyping. Proceedings of the National Academy of Sciences, 102, 14694–14699. 10.1073/PNAS.0507400102 PMC1253605 16203994

[tpg270066-bib-0064] Stich, B. , & Van Inghelandt, D. (2018). Prospects and potential uses of genomic prediction of key performance traits in tetraploid potato. Frontiers in Plant Science, 9, Article 159. 10.3389/fpls.2018.00159 29563919 PMC5845909

[tpg270066-bib-0065] Sverrisdóttir, E. , Byrne, S. , Sundmark, E. H. R. , Johnsen, H. Ø. , Kirk, H. G. , Asp, T. , Janss, L. , & Nielsen, K. L. (2017). Genomic prediction of starch content and chipping quality in tetraploid potato using genotyping‐by‐sequencing. Theoretical and Applied Genetics, 130, 2091–2108. 10.1007/s00122-017-2944-y 28707250 PMC5606954

[tpg270066-bib-0066] Sverrisdóttir, E. , Sundmark, E. H. R. , Johnsen, H. Ø. , Kirk, H. G. , Asp, T. , Janss, L. , Bryan, G. , & Nielsen, K. L. (2018). The value of expanding the training population to improve genomic selection models in tetraploid potato. Frontiers in Plant Science, 9, Article 1118. 10.3389/fpls.2018.01118 30131817 PMC6090097

[tpg270066-bib-0067] Terres, L. R. , Lenz, E. A. , Rocha, D. , Cerioli, M. , & Pereira, A. D. S. (2017). Combining ability of potato parents for tuber appearance and tuber yield component traits. Crop Breeding and Applied Biotechnology, 17, 99–106. 10.1590/1984-70332017V17N2A16

[tpg270066-bib-0068] Tong, C. , Ma, Z. , Chen, H. , & Gao, H. (2023). Toward an understanding of potato starch structure, function, biosynthesis, and applications. Food Frontiers, 4, 980–1000. 10.1002/fft2.223

[tpg270066-bib-0069] Urbany, C. , Stich, B. , Schmidt, L. , Simon, L. , Berding, H. , Junghans, H. , Niehoff, K.‐H. , Braun, A. , Tacke, E. , Hofferbert, H.‐R. , Lübeck, J. , Strahwald, J. , & Gebhardt, C. (2011). Association genetics in *Solanum tuberosum* provides new insights into potato tuber bruising and enzymatic tissue discoloration. BMC Genomics, 12, Article 7. 10.1186/1471-2164-12-7 21208436 PMC3023753

[tpg270066-bib-0070] Van Berloo, R. , Hutten, R. C. B. , Van Eck, H. J. , & Visser, R. G. F. (2007). An online potato pedigree database resource. Potato Research, 50, 45–57. 10.1007/S11540-007-9028-3

[tpg270066-bib-0071] Van Dijk, M. , Morley, T. , Rau, M. L. , & Saghai, Y. (2021). A meta‐analysis of projected global food demand and population at risk of hunger for the period 2010–2050. Nature Food, 2, 494–501. 10.1038/s43016-021-00322-9 37117684

[tpg270066-bib-0072] van Eck, H. J. (2007). Genetics of morphological and tuber traits. In D. Vreugdenhil (Ed.), Potato biology and biotechnology: Advances and perspectives (1st ed., pp. 91–115). Elsevier.

[tpg270066-bib-0073] Van Eck, H. J. , Jacobs, J. M. , Stam, P. , Ton, J. , Stiekema, W. J. , & Jacobsen, E. (1994). Multiple alleles for tuber shape in diploid potato detected by qualitative and quantitative genetic analysis using RFLPs. Genetics, 137, 303–309. 10.1093/GENETICS/137.1.303 7914504 PMC1205946

[tpg270066-bib-0074] VanRaden, P. M. (2008). Efficient methods to compute genomic predictions. Journal of Dairy Science, 91, 4414–4423. 10.3168/JDS.2007-0980 18946147

[tpg270066-bib-0075] Wickham, H. (2016). ggplot2: Elegant graphics for data analysis. Springer‐Verlag.

[tpg270066-bib-0076] Wilson, S. , Zheng, C. , Maliepaard, C. , Mulder, H. A. , Visser, R. G. F. , Van Der Burgt, A. , & Van Eeuwijk, F. (2021). Understanding the effectiveness of genomic prediction in tetraploid potato. Frontiers in Plant Science, 12, Article 672417. 10.3389/fpls.2021.672417 34434201 PMC8381724

[tpg270066-bib-0077] Wu, S. , Zhang, B. , Keyhaninejad, N. , Rodríguez, G. R. , Kim, H. J. , Chakrabarti, M. , Illa‐Berenguer, E. , Taitano, N. K. , Gonzalo, M. J. , Díaz, A. , Pan, Y. , Leisner, C. P. , Halterman, D. , Buell, C. R. , Weng, Y. , Jansky, S. H. , Van Eck, H. , Willemsen, J. , Monforte, A. J. , … Van Der Knaap, E. (2018). A common genetic mechanism underlies morphological diversity in fruits and other plant organs. Nature Communications, 9(1), Article 4734. 10.1038/s41467-018-07216-8 PMC6226536 30413711

[tpg270066-bib-0078] Wu, W. , Yu, Q. , You, L. , Chen, K. , Tang, H. , & Liu, J. (2018). Global cropping intensity gaps: Increasing food production without cropland expansion. Land Use Policy, 76, 515–525. 10.1016/j.landusepol.2018.02.032

