# Supplementary material for: The transmission ability in a population of elite tetraploid potatoes
Source: Plant Genome. 2025 Jun 30;18(3):e70066. doi: 10.1002/tpg2.70066 (PMC12207579; doi:10.1002/tpg2.70066)
Supplement: Supplementary file 1 [file TPG2-18-e70066-s001.docx]

The transmission ability in a population of elite tetraploid potatoes

Trine Aalborg^1*^, Hélène Romé^2^, Christina Ranzau^3^, Merethe Bagge^3^, Just Jensen^2^, Kåre Lehmann Nielsen^1,4^

^1^Department of Chemistry and Bioscience, Aalborg University, Aalborg, Denmark

^2^Center for Quantitative Genetics and Genomics, Aarhus University, Aarhus, Denmark

^3^Danespo A/S, Dyrskuevej 15, DK-7323 Give, Denmark

^4^KMC Amba, Denmark, Herningvej 60, DK-7330 Brande, Denmark

*Correspondence:
Trine Aalborg
[traa@bio.aau.dk](mailto:traa@bio.aau.dk)

**22 pages, 28 figures**

Supplementary figures

**Supplementary Figure S1**. Pedigree of MASPOT population parents (female progenitor in top lines). Created with Biorender.com.

**Supplementary Figure S2**. Heatmap of **A** matrix of the full MASPOT population (5013 F_1_ clones) and the 18 MASPOT parents.

**Supplementary Figure S3**. Double reduction rate included in the **A**-matrix against the relative -2log(L) (the difference relative to the recorded minimum value) of the linear mixed model fitted for dry matter content using the full MASPOT population (PBLUP_pop_).

**Supplementary Figure S4**. Heatmap of genomic relationship (**G**) matrix of 755 genotyped MASPOT F_1_ clones and the 18 MASPOT parents

**Supplementary Figure S5.** Single-step covariance (**H**) matrix of combined **A** and adjusted **G** matrix.

**Supplementary Figure S6.** QQ plots of the mean corrected phenotypes of the PBLUP_pop_ model. While some phenotypes are distributed most along the theoretical quantiles, according to Shapiro Wilks test on 5000 randomly sampled residuals for each trait model, none of the traits follow a normal distribution.

**Supplementary Figure S7.** Scaled additive general combining ability of MASPOT parents for all traits (with 95 % confidence interval) estimated with ssGBLUP.

**Supplementary Figure S8.** Non-additive specific combining ability of MASPOT families for dry matter content [%] estimated with PBLUP_pop_. Crosses with non-significant SCA estimates are shown in gray, and crosses without SCA contribution (SCA = 0) or infertile crosses are shown in white.

**Supplementary Figure S9.** Non-additive specific combining ability of MASPOT families for yield [hkg/ha] estimated with PBLUP_pop_. Crosses with non-significant SCA estimates are shown in gray, and crosses without SCA contribution (SCA = 0) or infertile crosses are shown in white.

**Supplementary Figure S10.** Non-additive specific combining ability of MASPOT families for eye depth [1-6 scale] estimated with PBLUP_pop_. Crosses with non-significant SCA estimates are shown in gray, and crosses without SCA contribution (SCA = 0) or infertile crosses are shown in white.

**Supplementary Figure S11.** Non-additive specific combining ability of MASPOT families for flesh color [1-9 scale] estimated with PBLUP_pop_. Crosses with non-significant SCA estimates are shown in gray, and crosses without SCA contribution (SCA = 0) or infertile crosses are shown in white.

**Supplementary Figure S12.** Non-additive specific combining ability of MASPOT families for skin finish [1-6 scale] estimated with PBLUP_pop_. Crosses with non-significant SCA estimates are shown in gray, and crosses without SCA contribution (SCA = 0) or infertile crosses are shown in white.

**Supplementary Figure S13.** Non-additive specific combining ability of MASPOT families for senescence [1-9 scale] estimated with PBLUP_pop_. Crosses with non-significant SCA estimates are shown in gray, and crosses without SCA contribution (SCA = 0) or infertile crosses are shown in white.

**Supplementary Figure S14.** Non-additive specific combining ability of MASPOT families for tubers/plant [tuber count] estimated with PBLUP_pop_. Crosses with non-significant SCA estimates are shown in gray, and crosses without SCA contribution (SCA = 0) or infertile crosses are shown in white.

**Supplementary Figure S15.** Non-additive specific combining ability of MASPOT families for length [mm] estimated with PBLUP_pop_. Crosses with non-significant SCA estimates are shown in gray, and crosses without SCA contribution (SCA = 0) or infertile crosses are shown in white.

**Supplementary Figure S16.** Non-additive specific combining ability of MASPOT families for diameter [mm] estimated with PBLUP_pop_. Crosses with non-significant SCA estimates are shown in gray, and crosses without SCA contribution (SCA = 0) or infertile crosses are shown in white.

**Supplementary Figure S17.** Non-additive specific combining ability of MASPOT families for length/width ratio estimated with PBLUP_pop_. Crosses with non-significant SCA estimates are shown in gray, and crosses without SCA contribution (SCA = 0) or infertile crosses are shown in white.

**Supplementary Figure S18.** Distribution of EBVs as a function of mean corrected phenotype (y_c_) for a repeat of the 8-fold random cross-validated ssGBLUP model. EBVs and $\bar{y_{c}}$ are in the units of the respective trait phenotype. The dispersoin bias was estimates as the slope of the linear regression line.

**Supplementary Figure S19.** Pearson prediction correlation coefficients (p($\bar{y_{c}}$,â_R_)) between mean corrected phenotypes ($\bar{y_{c}}$) and EBVs (â_R_) for 30 repeats of random 8-fold cross-validation for ssGBLUP based on H-matrices with based on G matrices of 150, 350, 550, and 755 genotyped clones and the full MASPOT population A matrix for dry matter content and yield.

**Supplementary Figure S20.** Additive general combining ability of MASPOT parents for yield [hkg/ha] (with 95 % confidence interval) estimated with PBLUP_pop_, PBLUP_panel_, GBLUP, and ssGBLUP.

**Supplementary Figure S21.** Additive general combining ability of MASPOT parents for eye depth [1-6 scale] (with 95 % confidence interval) estimated with PBLUP_pop_, PBLUP_panel_, GBLUP, and ssGBLUP.

**Supplementary Figure S22.** Additive general combining ability of MASPOT parents for flesh color [1-9 scale] (with 95 % confidence interval) estimated with PBLUP_pop_, PBLUP_panel_, GBLUP, and ssGBLUP

**Supplementary Figure S23.** Additive general combining ability of MASPOT parents for skin finish [1-6 scale] (with 95 % confidence interval) estimated with PBLUP_pop_, PBLUP_panel_, GBLUP, and ssGBLUP.

**Supplementary Figure S24.** Additive general combining ability of MASPOT parents for senescence [1-9 scale] (with 95 % confidence interval) estimated with PBLUP_pop_, PBLUP_panel_, GBLUP, and ssGBLUP.

**Supplementary Figure S25.** Additive general combining ability of MASPOT parents for tubers/plant [tuber count] (with 95 % confidence interval) estimated with PBLUP_pop_, PBLUP_panel_, GBLUP, and ssGBLUP

**Supplementary Figure S26.** Additive general combining ability of MASPOT parents for length [mm] (with 95 % confidence interval) estimated with PBLUP_pop_, PBLUP_panel_, GBLUP, and ssGBLUP.

**Supplementary Figure S27.** Additive general combining ability of MASPOT parents for diameter [mm] (with 95 % confidence interval) estimated with PBLUP_pop_, PBLUP_panel_, GBLUP, and ssGBLUP.

**Supplementary Figure S28.** Additive general combining ability of MASPOT parents for length/width ratio (with 95 % confidence interval) estimated with PBLUP_pop_, PBLUP_panel_, GBLUP, and ssGBLUP
