# Supplementary material for: The transmission ability in a population of elite tetraploid potatoes
Source: Plant Genome. 2025 Jun 30;18(3):e70066. doi: 10.1002/tpg2.70066 (PMC12207579; doi:10.1002/tpg2.70066)
Supplement: Supplementary file 2 [file TPG2-18-e70066-s002.docx]

The transmission ability in a population of elite tetraploid potatoes

Trine Aalborg^1*^, Hélène Romé^2^, Christina Ranzau^3^, Merethe Bagge^3^, Just Jensen^2^, Kåre Lehmann Nielsen^1,4^

^1^Department of Chemistry and Bioscience, Aalborg University, Aalborg, Denmark

^2^Center for Quantitative Genetics and Genomics, Aarhus University, Aarhus, Denmark

^3^Danespo A/S, Dyrskuevej 15, DK-7323 Give, Denmark

^4^KMC Amba, Denmark, Herningvej 60, DK-7330 Brande, Denmark

*Correspondence:
Trine Aalborg
[traa@bio.aau.dk](mailto:traa@bio.aau.dk)

**3 pages, 4 tables**

Supplementary tables

**Supplementary Table S1** Statistics describing the mean corrected phenotypic data across all years using the PBLUP_pop_ model: Range, mean ± standard error, median, phenotypic variance, coefficient of variance (CV), number of observations, number of replicates

| **Phenotype** | **Range** | **Mean (± S.E.)** | **Median** | **Variance** | **CV** | **No. obs.** | **No. rep.** |
| --- | --- | --- | --- | --- | --- | --- | --- |
| **Dry matter content** | (-13.10) – 36.84 | 0.05 ± 0.04 | 0.01 | 7.28 | 52.25 | 13,481 | 3 |
| **Yield** | (-657.44) – 1465.66 | 4.06 ± 2.56 | 7.70 | 32299.44 | 44.31 | 8,562 | 2 |
| **Eye depth** | (-2.33) – 2.42 | 0.00 ± 0.01 | (-0.01) | 0.55 | (-184.16) | 4,914 | 1 |
| **Flesh color** | (-4.28) – 2.90 | 0.08 ± 0.02 | 0.37 | 1.71 | 16.27 | 8,551 | 2 |
| **Skin finish** | (-2.09) – 1.76 | (-0.01) ± 0.01 | 0.06 | 0.44 | (-48.19) | 8,560 | 2 |
| **Senescence** | (-4.90) – 3.73 | (-0.01) ± 0.02 | 0.09 | 1.80 | (-97.34) | 8,570 | 2 |
| **Tubers/plant** | (-16.30) – 28.91 | 0.10 ± 0.08 | 0.02 | 30.12 | 52.35 | 8,524 | 2 |
| **Length** | (-40.21) – 70.93 | (-0.67) ± 0.14 | (-1.24) | 93.62 | (-14.38) | 8,433 | 2 |
| **Diameter** | (-27.13) – 27.34 | 0.03 ± 0.09 | 0.24 | 42.72 | 237.24 | 8,433 | 2 |
| **Length/width ratio** | (-0.35) – 1.25 | (-0.01) ± 0.00 | (-0.06) | 0.03 | (-14.91) | 8,433 | 2 |
| Eye depth: low = deep, high = shallow  Flesh color: low = white, high = orange  Skin finish: low = rough, high = smooth  Senescence: low = early, high = late | | | | | | | |

**Supplementary Table S2** Mean Pearson prediction correlation coefficient between predicted EBV in test set (â_R_) and mean corrected phenotype of 30 repeats of random 8-fold cross-validations ± standard deviation (s.d.), dispersion bias, narrow-sense heritability (plot and design), and prediction accuracy based on PBLUP_red_ model. The prediction accuracy is the correlation between the true breeding value (a) and the EBV

| **Trait** | **p(**$\bar{y_{c}}$**, â_R_) ± s.d.** | **Dispersion (**$\bar{y_{c}}$**, â_R_)** | **h^2^** | $\boldsymbol{h}_{\bar{\boldsymbol{y}_{\boldsymbol{c}}}}^{\boldsymbol{2}}$ | **p(a, â_R_)** |
| --- | --- | --- | --- | --- | --- |
| **Dry matter content** | 0.63 ± 0.01 | 1.07 | 0.40 | 0.42 | 0.95 |
| **Yield** | 0.21 ± 0.01 | 1.33 | 0.11 | 0.13 | 0.57 |
| **Eye depth** | 0.36 ± 0.01 | 1.11 | 0.51 | 0.51 | 0.53 |
| **Flesh color** | 0.49 ± 0.005 | 1.04 | 0.47 | 0.55 | 0.66 |
| **Skin finish** | 0.20 ± 0.01 | 1.08 | 0.12 | 0.17 | 0.47 |
| **Senescence** | 0.18 ± 0.01 | 1.13 | 0.09 | 0.11 | 0.56 |
| **Tubers/plant** | 0.33 ± 0.01 | 1.09 | 0.25 | 0.31 | 0.58 |
| **Length** | 0.48 ± 0.01 | 1.07 | 0.46 | 0.53 | 0.66 |
| **Diameter** | 0.31 ± 0.01 | 1.10 | 0.23 | 0.26 | 0.60 |
| **Length/width ratio** | 0.33 ± 0.01 | 1.15 | 0.24 | 0.28 | 0.59 |

**Supplementary Table S3** Mean pearson prediction correlation coefficient between predicted EBV in test set (â_R_) and mean corrected phenotype of 30 repeats of random 8-fold cross-validations ± standard deviation (s.d.), dispersion bias, narrow-sense heritability (plot and design), and prediction accuracy based on PBLUP_full_ model. The prediction accuracy is the correlation between the true breeding value (a) and the EBV

| **Trait** | **p(**$\bar{y_{c}}$**, â_R_) ± s.d.** | **Dispersion (**$\bar{y_{c}}$**, â_R_)** | **h^2^** | $\boldsymbol{h}_{\bar{\boldsymbol{y}_{\boldsymbol{c}}}}^{\boldsymbol{2}}$ | **p(a, â_R_)** |
| --- | --- | --- | --- | --- | --- |
| **Dry matter content** | 0.62 ± 0.001 | 1.05 | 0.30 | 0.32 | 1.11 |
| **Yield** | 0.25 ± 0.001 | 1.15 | 0.13 | 0.16 | 0.63 |
| **Eye depth** | 0.21 ± 0.003 | 1.24 | 0.13 | 0.13 | 0.56 |
| **Flesh color** | 0.50 ± 0.001 | 1.02 | 0.49 | 0.58 | 0.66 |
| **Skin finish** | 0.29 ± 0.002 | 1.06 | 0.15 | 0.20 | 0.65 |
| **Senescence** | 0.22 ± 0.002 | 1.00 | 0.09 | 0.11 | 0.64 |
| **Tubers/plant** | 0.32 ± 0.001 | 1.07 | 0.22 | 0.26 | 0.63 |
| **Length** | 0.50 ± 0.001 | 1.02 | 0.54 | 0.61 | 0.64 |
| **Diameter** | 0.33 ± 0.001 | 1.11 | 0.23 | 0.26 | 0.65 |
| **Length/width ratio** | 0.40 ± 0.001 | 1.10 | 0.34 | 0.37 | 0.67 |

**Supplementary Table S4** Mean pearson prediction correlation coefficient between predicted EBV in test set (â_R_) and mean corrected phenotype of 30 repeats of random 8-fold cross-validations ± standard deviation (s.d.), dispersion bias, narrow-sense heritability (plot and design), and prediction accuracy based on GBLUP model. The prediction accuracy is the correlation between the true breeding value (a) and the EBV

| **Trait** | **p(**$\bar{y_{c}}$**, â_R_) ± s.d.** | **Dispersion (**$\bar{y_{c}}$**, â_R_)** | **h^2^** | $\boldsymbol{h}_{\bar{\boldsymbol{y}_{\boldsymbol{c}}}}^{\boldsymbol{2}}$ | **p(a, â_R_)** |
| --- | --- | --- | --- | --- | --- |
| **Dry matter content** | 0.71 ± 0.004 | 1.09 | 0.34 | 0.37 | 1.15 |
| **Yield** | 0.24 ± 0.02 | 1.25 | 0.18 | 0.22 | 0.50 |
| **Eye depth** | 0.29 ± 0.02 | 1.13 | 0.38 | 0.38 | 0.51 |
| **Flesh color** | 0.67 ± 0.01 | 1.08 | 0.63 | 0.77 | 0.76 |
| **Skin finish** | 0.23 ± 0.01 | 1.04 | 0.18 | 0.25 | 0.44 |
| **Senescence** | 0.31 ± 0.01 | 1.04 | 0.35 | 0.42 | 0.48 |
| **Tubers/plant** | 0.39 ± 0.01 | 1.08 | 0.32 | 0.40 | 0.60 |
| **Length** | 0.53 ± 0.01 | 1.08 | 0.50 | 0.59 | 0.66 |
| **Diameter** | 0.38 ± 0.01 | 1.07 | 0.42 | 0.48 | 0.52 |
| **Length/width ratio** | 0.44 ± 0.01 | 1.10 | 0.52 | 0.62 | 0.54 |
